# Spatiotemporal patterns of lower-band whistler mode waves in the magnetosphere of Earth

**DOI:** 10.1038/s41467-026-75552-1

**Published:** 2026-07-30

**Authors:** O. Santolík, I. Kolmašová, U. Taubenschuss, M. Hanzelka

**Affiliations:** 1 Department of Space Physics, Institute of Atmospheric Physics of the Czech Academy of Sciences, Prague, Czechia; 2 Faculty of Mathematics and Physics, Charles University, Prague, Czechia

**Keywords:** Magnetospheric physics, Space physics

## Abstract

Electromagnetic emissions of lower-band chorus and exohiss waves are known to interact with electrons in the Earth’s outer radiation belt. Understanding statistical properties of these natural emissions is essential for modeling space weather hazards and protecting satellite infrastructure. Despite extensive previous studies, their spatiotemporal characteristics remain incompletely understood. Here, we analyze a large data set of spacecraft measurements, showing that distributions of wave amplitudes exhibit a wide log-normal core with an irregular heavy tail, changing with geomagnetic activity and location. The distribution of time intervals between wave detections follows a power law, suggestive of temporal bunching. Around local noon, we nearly always detect waves up to high latitudes, while intense waves occur for less than a few percent of the time, predominantly in the postmidnight equatorial region. Our findings indicate that the burstiness of whistler-mode waves may not be fully captured by long-term averages, which are commonly used in radiation belt modeling.

## Abstract (web summary)

Natural electromagnetic emissions at audible frequencies strongly affect the outer radiation belt. Here, the authors show that their occurrence is clustered in time and follows a distinct spatial pattern in both the magnetic local time and latitude.

## Introduction

The outer Van Allen radiation belt experiences significant effects of whistler mode wave emissions^1^, which can rapidly intensify electron fluxes at relativistic energies^2–4^ or precipitate them into the atmosphere^5–8^. In the magnetospheric regions outside of the high-density plasmasphere, these electromagnetic waves occur in the form of chorus^9,10^, consisting of discrete chirps in the audible frequency range, but also in the form of hiss without distinct spectral features. Practical importance of their investigation is demonstrated by the need of operational radiation forecasting in the outer Van Allen belt^11,12^ with the societal implications for safety of space assets^13^.

Chorus waves can also propagate down to the ground. According to the initial description after the discovery of these emissions in ground-based measurements, sound of dawn chorus may be likened to that of a rookery heard from the distance; it consists of a multitude of rising whistles against a background of a warbling sound which may be mixed with varying amounts of toneless hissing^14^. This mixture of different spectral features is also a characteristic property of whistler mode emissions observed later by spacecraft in the magnetosphere of Earth^10,15–17^. Forecasting models of particle fluxes use a quasi-linear approach, in which both exohiss and chorus of the same amplitude propagating in the same plasma medium have the same effects on energetic particles^12,18–20^, neglecting non-linear effects of discrete frequency-time structures in chorus elements or in their subpackets^21–23^.

These empirical models^24–32^ are based on regularly sampled low resolution measurements at different locations and under different conditions, which are analyzed onboard the spacecraft^33–35^. The datasets are collected over long time periods, which are comparable to the operational periods of spacecraft missions. The measurements are binned in predefined intervals of spatial coordinates and geomagnetic activity indices or solar wind parameters. A crucial parameter for the quasi-linear approach is the squared wave amplitude, and its bin-average values serve in these models to estimate the diffusion coefficients and determine particle fluxes. The distributions of squared amplitudes are known from the observations^26,36^ to be highly dispersed and heavy-tailed. The necessary assumption of existing quasilinear models is that their long-term averages obtained on the time scales of operational periods of spacecraft missions are also representative for much shorter temporal scales of the particle acceleration and loss processes, which can be as short as several hours^2–4^.

The data set of lower-band whistler-mode chorus and exohiss with instrumental noise thresholds^37^ combines almost 7 years of Survey data measurements by the EMFISIS Waves instruments^33,38^ on two low-latitude Van Allen Probes spacecraft, with more than 19 years of Normal mode data of the STAFF-SA instruments^34,39^ from all four high-inclination Cluster spacecraft. The data set is cleaned from measurement artifacts and uses carefully characterized frequency dependent detection thresholds and their evolution during the lifetime of the two spacecraft missions to separate the detections of natural waves from instrumental noise.

Here, we show, taking advantage of the database^37^, what are the main characteristics of spatio-temporal distributions of all detectable events of lower-band chorus and exohiss as a function of position within the magnetosphere and time delays between the detections. Weak waves are nearly always present in the noon sector over a large range of geomagnetic latitudes, being amplified during their propagation from the equator. The time intervals between detections have, in general, a power-law distribution, which reflects strong clustering of their occurrences. Very intense waves above 300 pT, which contribute to the long-term average of squared amplitudes in their dawn-side low-latitude peak are observed during less than 0.5 % of time, with average time intervals of nearly 10 h between detections in our data set. This is comparable to the possible time scales of quasilinear diffusion of the radiation belt electrons. Therefore, the long-term averages of squared amplitudes may not always be representative for description of chorus-exohiss waves involved in these processes.

## Results

### Distributions of squared amplitudes

**Fig. 1:**
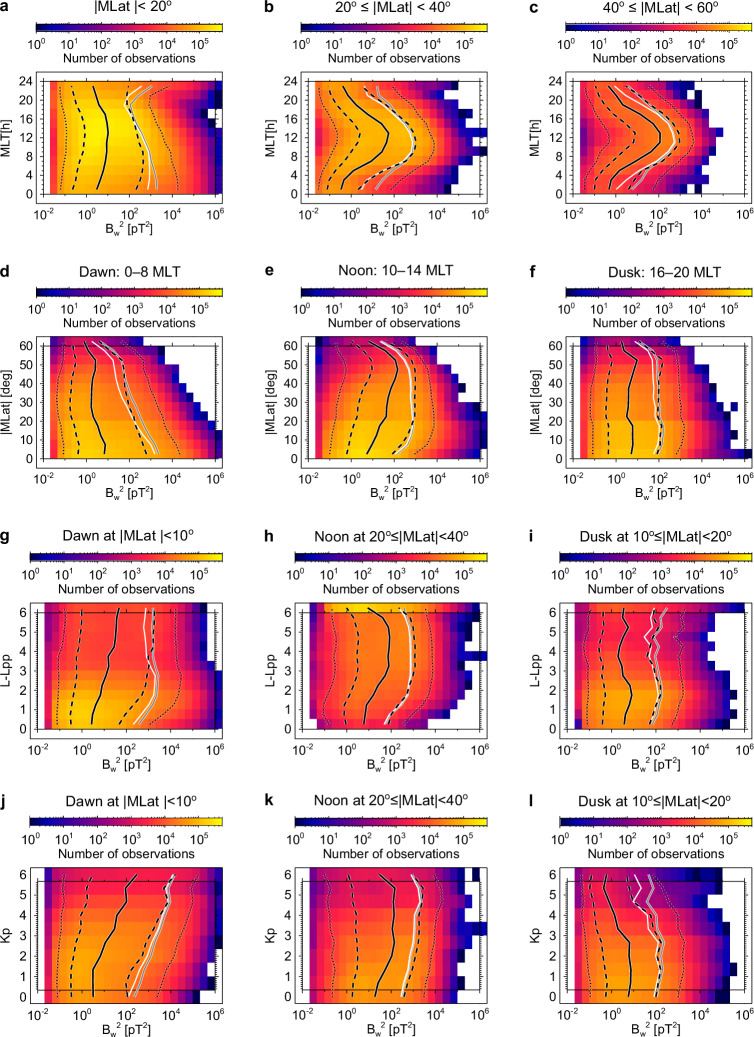
Distribution of squared amplitudes of lower band chorus/exohiss. **a***–***c** Set of histograms of the frequency integrated trace of the magnetic power spectral density matrix *B*_w_^2^ in 12 discrete bins of magnetic local time MLT, for three intervals of the absolute value of magnetic latitude: **a** |MLat|<20°, **b** 20° ≤ |MLat | <40°, **c** 40° ≤ |MLat | <60°; **d***–***f** in 13 discrete bins of |MLat|, **f**or three intervals of MLT: **d** Dawn: 0–8 MLT, **e** Noon: 10–14 MLT, **f** Dusk: 16–20 MLT; **g***–***i** in 13 discrete bins of the equatorial distance (in Earth’s radii) from the model plasmapause^40^ *L* − *L*_PP_ for three intervals of MLT and |MLat|: **g** 0–8 MLT at |MLat|<10°, **h** 10–14 MLT at 20° ≤ |MLat | <40°, **i** 16–20 MLT at 10° ≤ |MLat | < 20°. **j-l** in 10 discrete b**i**ns of the geomagnetic activity index *K*_P_, for the same intervals of MLT and |MLat | , as in panels g*–*i. The boundary bins between the two black rectangles on each plot collect all data falling outside the plotting range. The median value *Q*_*b50*_ of *B*_w_^2^ from Eq. (9) is over-plotted by a black solid line, 15.9 and 84.1 percentiles (±1 standard deviation from the median for a normal distribution) by dashed lines, 2.3 and 97.7 percentiles (±2 standard deviations from the median for a normal distribution) by dotted lines; the estimated mean value *B*_*b*_ of *B*_w_^2^ from Eq. (12) is given by a gray solid line, the white solid line shows the long-term average *r*_*b*_ *B*_*b*_, corresponding to the mean value *B*_*b*_ normalized by the occur*r*ence rate *r*_*b*_ from Eq. (6). Source data are provided as a Source Data file.

Distributions of obtained three-dimensional squared amplitudes of magnetic field fluctuations *B*_w_^2^ of detected chorus/exohiss emissions are shown in Fig. 1a–c as a function of the magnetic local time (MLT) for three intervals of the absolute value of the magnetic latitude |MLat|. The *B*_w_^2^ values are calculated from the traces of the spectral density matrices obtained by three-axial magnetic search coil measurements and integrated over the lower-band frequency range from 0.1 to 0.5 of the equatorial electron cyclotron frequency. Their distribution is characterized by the estimates of the median value and four additional percentiles, by the estimate of the mean value, and by the “long-term average” obtained as a product of the mean value and occurrence probability (see Methods). A color-coded background shows the number of measurements in each MLT—*B*_w_^2^ bin. The distributions are very wide, spanning over several orders of magnitude of *B*_w_^2^. Their core parts often approximately follow the log-normal shape, with roughly equal distance of the “one-sigma” percentiles from the median. The mean value of this heavy tail distribution is often close to the 84.1 percentile, more than one order of magnitude above the median value.

All characteristics of these distributions demonstrate a strong dependence on MLT. In the equatorial region with |MLat| below 20° (Fig. 1a) the median value (solid black line) shows a flat peak around 10 pT^2^ on the dayside but the mean value (gray line) peaks around midnight near 1900 pT^2^, as the width of the distribution increases. This effect is amplified by a pronounced deviation of the distribution from the log-normal shape toward an even heavier tail at high *B*_w_^2^, reaching occasionally above 1 nT^2^. The long-term average (white line in Fig. 1a) has a flat peak of nearly 900 pT^2^, which is shifted toward dawn due to a decrease in wave occurrence around midnight. Pronounced minima of the mean and long-term average values (down to 60 pT^2^) are observed in the dusk sector at 16–20 MLT. At higher latitudes (Fig. 1b, c), a very pronounced peak around local noon (10–14 MLT) is observed in all the characteristics of the *B*_w_^2^ distribution, with a distribution closer to a log-normal shape, median values increasing with latitude, and long-term averages peaking above 700 pT^2^ in the noon sector.

Figure 1d–f shows details of the *B*_w_^2^ distribution characteristics as a function of latitude in the three above-mentioned interesting narrower MLT sectors. The results in Fig. 1d for the dawn side (0–8 MLT) confirm the non-lognormality of the distribution in the equatorial region, leading to a heavy tail and hence high values of the mean and long-term average values (2300 pT^2^ and 1500 pT^2^, respectively) peaking within 5° from the equator, while keeping the median value below 10 pT^2^. The heavy tail feature of the distribution then progressively weakens toward higher latitudes with slightly decreasing median values, causing a rapid decrease in higher percentiles of the distribution, as well as the mean and long-term average values. A very different behavior is observed in Fig. 1e for the noon sector (10–14 MLT), where the median value increases with latitude up to 50°, reaching 150 pT^2^, and the long-term average grows up to a latitude of 20° and then it stays approximately constant around 600–750 pT^2^. In the dusk sector at 16–20 MLT (Fig. 1f), all the characteristics stay approximately constant with latitude up to 50°, with the long-term average at 60–100 pT^2^. Note that these values (shown in Fig. 1 by the white lines) are relevant for the long-term radiation belt quasi-linear simulations.

As all the previous results were accumulated at equatorial distances of the field line from the model plasmapause *L* − *L*_PP_ (see Methods) between 1 and 6 Earth’s radii (*R*_E_), it is important to verify the evolution of the *B*_w_^2^ distribution as a function of *L* − *L*_PP_. Figure 1g–i shows these results in three interesting regions: in the equatorial dawn sector at latitudes below 10° (Fig. 1g), in the noon sector above 20° (Fig. 1e), and in the dusk sector between 10° and 20°. In all three regions, we observe an initial increase of all the distribution characteristics within 2 *R*_E_ from the model plasmapause, followed by a weak variation of the long-term average values, and a continuing increase or weak variation of other distribution characteristics. The selection of the *L*−*L*_PP_ range, and the uncertainty of the *L*_PP_ model^40^, therefore, do not substantially alter the obtained results.

The geomagnetic activity level is also known for influencing chorus amplitudes^1,24,41,42^, and Fig. 1j–l show the evolution of *B*_w_^2^ distributions as a function of planetary activity index *K*_P_ in the same three regions of interest as in Fig. 1g–i. While all the characteristics of the distribution in the dawn equatorial region in Fig. 1j grow with *K*_P_ (for the long-term average value from 120 pT^2^ to 12,500 pT^2^ at *K*_P_ > 6–), the stochastic spread of the *B*_w_^2^ distribution between the 15.9 and 84.1 percentiles is always larger than this systematic variation. In the noon sector (Fig. 1k), the growth of the distribution characteristics with *K*_P_ is much slower^41^, and in the dusk sector (Fig. 1l), they may even decrease for higher *K*_P_ values.

Note that similar results as for the *L*−*L*_PP_ parameter are obtained as a simple function of the *L* parameter, which was found to order the chorus amplitudes^43^, and that the effects of geomagnetic activity are also very similar based on the auroral AL* index, often used to characterize the substorm-related sources of chorus^9,24^ and calculated as the minimum AL over the past 3 hours (Supplementary Fig. 1).

### Occurrence patterns

**Fig. 2:**
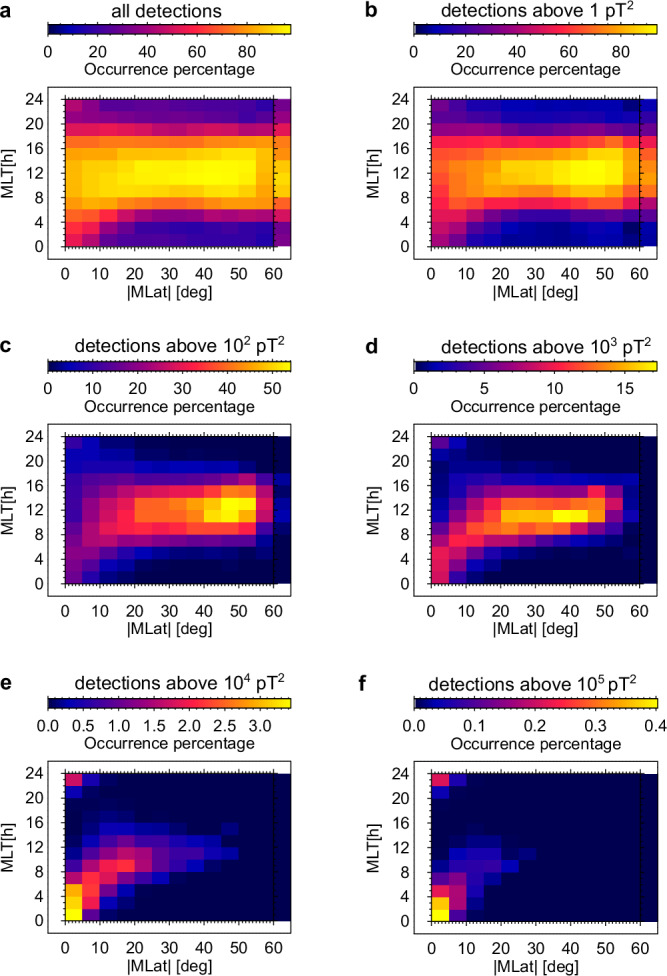
Spatial distribution of occurrence probabilities of lower band chorus/exohiss. **a** Occurrence percentage *R*_*b*_ from Eq. (7) is analyzed from a joint data set of two Van Allen Probes and four Cluster spacecraft in 12 × 13 discrete bins in magnetic local time MLT and absolute value of the magnetic latitude |MLat| up to 60°, for the equatorial distance from the plasmapause^40^ field line *L*−*L*_PP_ between 1 and 6 *R*_E_ but within the magnetopause^73^; summary results for latitudes above 60° are plotted on the right-hand side of the plot. **b** The same but with an additional constraint for the frequency-integrated trace of the magnetic power spectral density matrix to be larger than 1 pT^2^, **c** 10^2^ pT^2^, **d** 10^3^ pT^2^, **e** 10^4^ pT^2^, and **f** 10^5^ pT^2^. Source data are provided as a Source Data file.

Figure 2 shows a more comprehensive representation of the distributions from Fig. 1a–f. Analyzing all whistler mode waves in the frequency range of lower band chorus, we found that their global occurrence rates form a nearly symmetric pattern around the local noon meridian (Fig. 2a). The occurrence probability reaches above 80% on the dayside between 8 and 16 MLT, and in the entire analyzed range of absolute values of the magnetic latitude up to 60°. Within this range, the occurrence probability slightly increases from the equator up to a latitude of 20°, from where the peak occurrence probability above 95% extends to higher latitudes up to 50° in the noon sector between 10 and 14 MLT. Around midnight MLT, the occurrence rate goes down to 50% at low latitudes, smoothly decreasing to approximately 10% at higher latitudes.

The prevailing dayside occurrence pattern is necessarily dominated by lower amplitudes, considering the highly squeezed distribution of *B*_w_^2^ in Fig. 1. Figure 2b–f shows occurrence probabilities of plasmatrough whistler mode waves in the frequency range of lower band chorus with additional constraints. Not only must the trace of their magnetic field spectral matrices in the corresponding instrumental frequency channels be above the frequency and time-dependent detection thresholds, but also the frequency-integrated trace (i.e., the three-dimensional squared amplitude *B*_w_^2^) must be above a predefined value, ranging from 1 pT^2^ to 10^5^ pT^2^ in Fig. 2b–f. This corresponds to three-dimensional root-mean-square amplitudes from 1 pT to 316 pT.

**Fig. 3:**
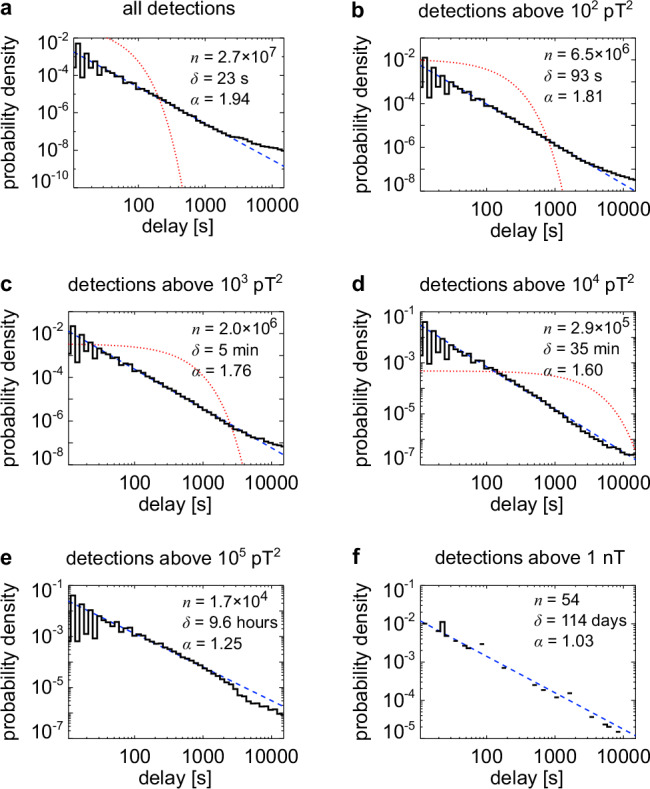
Global time delay statistics. **a** Probability density function of time delays between detections from Eq. (15), obtained using the same data set as in Fig. 2a, with a blue dashed line showing a power-law fit from Eq. (3) for delays between 30 and 2000 s, with the overwritten number of samples *n*, average delay *δ*, and power-law exponent *α*, and with a red dotted line showing an exponential distribution from Eq. (1) for the expected value *Δ* estimated by *δ*; **b–e** the same but for constrained data sets from Fig. 2c–f, respectively; **f** the same but with a constraint for the frequency integrated trace of the magnetic power spectral density matrix to be larger than 1 nT^2^. Source data are provided as a Source Data file.

This procedure, with an additional constraint of amplitudes larger than 1 pT returns similar results (Fig. 2b) as the original occurrence percentage in Fig. 2a, with the noon-centered peak reaching above 90% at latitudes between 35° and 55°, but the low-latitude occurrence probability on the dayside decreases down to 65% close to the equator. For the constraint of 10 pT (Fig. 2c) this high-latitude occurrence peak around local noon only reaches above 50%, while the equatorial occurrence probability decreases below 20% on the morning side and below 10% around local noon. A constraint of 31.6 pT (Fig. 2d) still shows the peak occurrence probability close to local noon at latitudes between 20° and 50°, with a maximum down to 17%, and with an occurrence probability around 10% in the postmidnight equatorial region. A constraint of 100 pT (Fig. 2e) moves the maximum occurrence probability to this latter region, reaching slightly above 3%. Finally, large amplitude waves for a constraint of 316 pT (Fig. 2f) confirm the confinement of occurrence to the postmidnight equatorial region, but with a peak probability of only 0.4%. Amplitudes with an additional constraint of 1 nT (used later in Fig. 3f) result in a small maximum probability of 10^−3^%.

This behavior is repeated also in different parts of the plasmatrough, for example if we restrict the analysis to a region close to the plasmapause (Supplementary Figs. 2a–f). The same picture of a symmetric peak with nearly 100% occurrence probability around local noon is also repeated at high latitudes in the outer regions of the plasmatrough (Supplementary Figs. 3a–f). Here, however, the low-latitude occurrence rates show a peak around 90% but slightly shifted from local noon to around 10 MLT (Supplementary Fig. 3a), while the constraint of large wave amplitudes (Supplementary Fig. 3e, f) moves the low-latitude maximum occurrence probability in the opposite direction, from the post midnight sector toward the morning side around 7 MLT.

The obtained nearly 100 % occurrence around local noon in a wide range of latitudes also demonstrates good stability in different phases of the Solar cycles 23 and 24 (Supplementary Fig. 4), noting that close to the solar maxima, the occurrence grows at low latitudes also in other MLT sectors. This is also observed for the additional constraint of wave amplitudes above 100 pT (Supplementary Fig. 5), for which the night side low latitude occurrence percentage substantially grows during the active phases of the Solar cycle.

Similar effects are observed when we select the data according to the geomagnetic substorm activity levels (Supplementary Fig. 6). A peak of nearly 100% occurrence centered around local noon in a wide range of latitudes above 20° is persistent for low and moderate activity levels, with decreasing occurrence toward the equator for low activity. For a small fraction (about 2%) of measurements during highly active times, the occurrence peak moves from local noon to 10 MLT. Intense waves with root-mean-square amplitudes above 100 pT occur in the post-midnight equatorial region substantially more often during moderately and highly active times, reaching occurrence rates above 10%.

### Temporal clustering of detections

With the same combined dataset from all 6 spacecraft, sorted according to the constraints imposed on the squared amplitude, we can analyze the temporal properties of the lower-band chorus/hiss emissions. If the detections of these waves correspond to a random process without memory, where detections occur independently of each other, we would expect a Poisson probability distribution for the number of detections that occur during a predefined time interval. This would be equivalent to the exponential probability density,

1 $${{{\rm{PDF}}}}_{{{\rm{E}}}}=\frac{1}{\Delta }\exp \left(-\frac{d}{\Delta }\right)$$

for time delay *d* between the subsequent detections, where Δ is the expected value for the delay.

Figure 3a shows a histogram of the probability density function of the time delay, as we experimentally determined it from Eq. (15) for all detected events, corresponding to the spatial distribution in Fig. 2a with predominantly low amplitudes and localized mainly on the dayside. The expected value of the delay Δ is estimated using the sample average as

2 $$\Delta \cong \delta=\frac{1}{n}{\sum }_{i=1}^{n}{d}_{i}$$

where *n* is the number of separate time delay measurements *d*_*i*_, obtaining in this case *δ* = 23 s. We can see that the observed probability density is far from the exponential function, which is shown for comparison by a red dotted line. The observed distribution is rather close to a truncated power-law function,

3 $${{{\rm{PDF}}}}_{{{\rm{P}}}}=k{d}^{-\alpha },$$

for *d* > *D*_0_, where *α* is an exponent, *k* is a normalization factor, and *D*_0_ is the minimum delay, given here by the measurement cadence (4 s for Cluster and 6 s for Van Allen Probes). However, for delays below 30 s, we still see pronounced effects of the finite measurement cadence. For delays between 30 and 2000 s, we obtain a fit of a power-law distribution with *α* = 1.94, shown by the blue dashed line, and resulting from mutual dependence and clustering of events at different temporal scales. The obtained power-law exponent is below 2, which in theory means that the expected value of the power-law distribution diverges, and the average value *δ* depends on *n*. However, the delays *d* also have an upper bound (defined by the spacecraft orbit, or, ultimately, by the total duration of the data set), and the average value *δ* still can be used as a measure of a typical time delay between the events across the given data set. The maximum displayed delay is 16,200 s, corresponding to one half of the Van Allen Probes orbital period, but already above delays of 2000 s the experimental histogram shows an even heavier tail of the distribution compared to the model fit.

Analysis with squared amplitudes constrained above 100 pT^2^ up to above 1 nT^2^ (Fig. 3b–f, respectively) shows that the average delay *δ* increases from 1.5 min to 114 days. This is accompanied by a decreasing exponent *α* from nearly 1.9 down to *α* ≈ 1. An important threshold is connected to the change in spatial distribution from the predominant dayside to the dawn/night side occurrence (in Figs. 2a–d and 2e–f, respectively), which gradually appears around a constraint of 10^4^ pT^2^. The power-law exponent of the time delay distribution linked to this change is *α* = 1.6, and the average delay *δ* is more than 30 min. For a 10 times larger constraint (10^5^ pT^2^) the exponent is *α* = 1.25, and *δ* is close to 10 h.

**Fig. 4:**
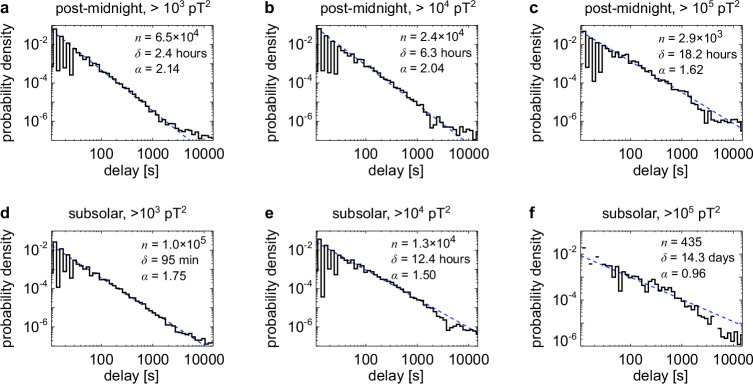
Localized time delay statistics. Probability density functions of time delays between detections from Eq. (15), obtained using the data sets from Fig. 2d–f, which are constrained by squared amplitude thresholds of 10^3^, 10^4^, and 10^5^ pT^2^, respectively. Additional constraints are used to localize the measurements in space into **a–c** post-midnight region at 0–2 MLT and *L* = 5–6; **d–f** subsolar region at 11–13 MLT and *L* = 5–6. Blue dashed lines show power-law fits from Eq. (3) for delays between 30 s and 2000 s, with an overwritten number of samples *n*, average delay *δ* and power-law exponent *α* for each fit. Source data are provided as a Source Data file.

As all the measurements are done by orbiting spacecraft, the global time-delay statistics in Fig. 3 also reflect the changing spacecraft location. We therefore further constrained the analyzed data set into two important regions: the post-midnight sector between midnight and 2 MLT (Fig. 4a–c), and the noon sector around the subsolar magnetic meridian between 11 and 13 MLT (Fig. 4d–f). In both cases, we additionally limited the analyzed *L* parameters between 5 and 6 but we didn’t include any latitudinal constraints. The time the spacecraft needed to pass through these regions in separate orbits strongly vary but are, on average, 9400s for Van Allen Probes, and 3900s for the Cluster spacecraft, with the maximum traversal times of 13,800 s and 5400 s, respectively. Delays above these limits are linked to the detections by a different spacecraft from our constellation entering the analyzed region later. However, owing to a limited total time spent by all the spacecraft in both regions in Fig. 4, the average delays *δ* are much longer than *δ* values obtained from the global data set in Fig. 3 for the same amplitude constraints.

Figure 4 clearly demonstrates that a truncated power-law function still is a good model for the probability density function of observed delays but that substantial differences in the temporal properties of the lower band chorus/hiss emissions occur in the two analyzed regions. While in the post-midnight sector the power-law exponent *α* stays close to 2 for the squared amplitude constraints of 10^3^ and 10^4^ pT^2^ and only decreases to 1.6 for the constraint of 10^5^ pT^2^, the noon sector exhibits a decreasing exponent from 1.75, down to a value close to 1 for the same constraints.

## Discussion

Our results show a clear separation of two populations of lower band chorus/exohiss as a function of wave amplitude threshold: (a) low-amplitude waves on the dayside, noon-centered, across all latitudes up to ±50° (b) high-amplitude waves, whose occurrence shifts sharply to the post-midnight equatorial region. This amplitude-dependent shift documented using a large data set^37^ directly constrains the two distinct generation regimes of these waves. Prior work^24,27,29,44^ had lower coverage and noted a dayside peak but could not resolve this amplitude-dependent transition from dayside to post-midnight dominance with the same precision. Our results also show a heavy-tail distribution of squared amplitudes of lower-band chorus/exohiss, having a large spread of 2–3.5 orders of magnitude between the 15.9 and 84.1 percentiles (Fig. 1). This inherent width of the distribution is not substantially reduced using parametrization by geomagnetic indices (Fig. 1j–l, Supplementary Fig. 1d–f), as it is linked to a bursty nature of these emissions, which is also reflected by the power-law distribution of time intervals between their detections. This distribution results from a large-scale statistical quantification of the inter-event time distribution as a function of amplitude and location. It reflects clustering across timescales over two orders of magnitude, implying that wave generation events are not independent but mutually correlated.

We confirm previous findings^9,10,24,44^, that the large amplitude waves above 100 pT tend to predominantly occur in the post-midnight sector (Fig. 2e) and at low latitudes within 5–10° of the geomagnetic equator but their occurrence probability is below 4%. We find that in these regions the amplitude distribution shows a significantly heavier tail than what would correspond to the log-normal shape (Fig. 2a, d), but that these large amplitude waves also have a power-law distribution of time delays between neighboring detections (Figs. 3d–f, 4a–c). The exponent of 1.6 is decreasing down to ≈1 when we increase the amplitude threshold from 100 pT up to 1 nT throughout the whole dataset (Fig. 3d–f) but stays close to 2 for the 100-pT threshold in a localized spatial region in the post-midnight sector (Fig. 4b). The average time delay between these events for the entire dataset is 30 min for the threshold of 100 pT, rapidly increasing to nearly 10 h at 316 pT, and to more than 100 days at 1 nT. The highly skewed power-law distribution, however, puts the median value much lower, to 6 s, 36 s, and 1.5 days, respectively, for these three thresholds.

The established generation mechanism of this class of high amplitude waves is linked to charged particles from the plasmasheet in the center of the magnetospheric tail, which can be injected during geomagnetic substorms into the nightside inner magnetosphere and adiabatically energized^45,46^. The clouds of energetic anisotropic electrons at energies of tens of keV then drift eastwards in the Earth’s magnetic field from the midnight sector through dawn to noon, while staying at low latitudes around the geomagnetic equator. Their anisotropy drives them into the cyclotron instability, which can generate electromagnetic chorus waves whose intensity increases with increasing geomagnetic activity^9,47^ in active phases of the Solar cycle, as we confirm in Supplementary Figs. 6d, and 6f.

When analyzing all detectable events, it means without the constraint of high amplitude waves, we show that chorus and exohiss waves occur during nearly 100% of time, and over a large range of latitudes in the noon-centered dayside region (Fig. 2a). Previous analysis of filter bank data from 20 months of low-latitude measurements of five THEMIS spacecraft^24^ found that occurrence rates of low-amplitude waves above 10 pT can reach 30–40% in the outer plasmatrough at *L* > 7, with a peak centered before noon, around 8–10 MLT. Another study based on 10 years of measurements of one of the Cluster spacecraft^44^ obtained an occurrence peak at latitudes above 15° in the afternoon sector, around 15–16 MLT for wave amplitudes above 1 pT. Our results globally agree with the previous results^24^ that the occurrence rate of the moderate (*B*_w_ < 30 pT) and strong (30 pT < *B*_w_ < 100 pT) dayside chorus is much larger than that on the nightside in both the near-equatorial region and at mid-latitudes. We find that the power-law distribution of time delays between neighboring detections of these waves has a larger exponent, decreasing from nearly 2 down to 1.6 (Fig. 3a–c). However, when we constrain the data set to higher amplitudes, the power-law exponent in a localized spatial region in the noon sector rapidly decreases (Fig. 4d–f).

The possibility that sources of high-latitude chorus can be found in local minima of the magnetic field strength on the dayside near the magnetopause has been previously discussed^47,48^. However, subsequent analysis of the Poynting flux from newer measurements^49–52^ showed that the contribution of chorus from the high latitude local minima to the total Poynting flux of chorus was minor, although we cannot exclude that chorus from the high latitude magnetic field minima contributes in part to the observed predominant dayside occurrence of chorus and exohiss. Origin of the dayside waves at low latitudes can be likely linked to the influence of solar wind pressure pulses on the dayside magnetosphere as a consequence of high-density heliospheric plasmasheet events^52,53^, leading subsequently to increased electron anisotropy and wave generation via the cyclotron instability in the noon sector or to a similar effect of natural enhancement of electron anisotropy around noon^24,54^.

We show that all the characteristics of the amplitude distributions of these dayside waves significantly grow with latitude up to about 20°, indicative of a convective growth in this region (Fig. 1e). While the median values keep growing up to about 50°, the long-term average values are approximately constant between 20° and 50°, owing to the decreasing width of the approximately log-normal distribution. Although the long-term average roughly agrees with previous statistics^24,27^, it is more accurate and available up to higher latitudes thanks to the larger dataset used here, which is important for radiation belt modeling^55^. The distribution in Fig. 1e is also consistent with the previously inferred^23,56–58^ apparent presence of significant chorus wave ducting in the noon sector.

The probability density distributions of time delays between neighboring lower band chorus/exohiss observations are consistently close to a power-law model in Figs. 3 and 4. This is valid for all analyzed amplitude thresholds and for both the global data set and data sets constrained to localized spatial regions. The power-law exponents *α* differ for these different combinations of constraints but always are nearly constant for delays between 30 s and 2000 s in each case. Below 30 s, all experimental histograms strongly fluctuate because of beating of their bin widths with the finite cadence of measurements in our data set. For delays longer than 2000 s, the obtained probability density function generally occurs above the power-law model when the amplitude threshold is lower than approximately 100 pT, or below it for larger thresholds.

The characteristic delay of 2000 s either reflects properties of temporal clustering of the analyzed chorus/exohiss emissions in a “global source region” extended over a region of several Earth radii^59^, or, for the dayside chorus/exohiss emissions, it corresponds to the limited spatial extent of a typical “chorus active region”, which was estimated to 4000–8000 km at 8–14 MLT in the transverse direction^60^. The transversal component of the orbital speed of Van Allen Probes and Cluster spacecraft in the plasmatrough substantially varies, but typical values are between 2 and 3 km/s. The spacecraft therefore should traverse the chorus active region in 1300–4000 s, which is roughly comparable to the characteristic delay of 2000 s, and provides thus an alternative explanation of our observations. Estimates of characteristic transverse sizes of sources of individual discrete chorus wave packets of 100 km^61–64^, or 400–800 km for typical transverse sizes of chorus source region^60,65^ give characteristic traversal times below 400 s. At these delays we do not observe any pronounced changes in the power-law statistics in Figs. 3 and 4, so these characteristic dimensions do not seem to determine the observed temporal properties of wave observations.

**Fig. 5:**
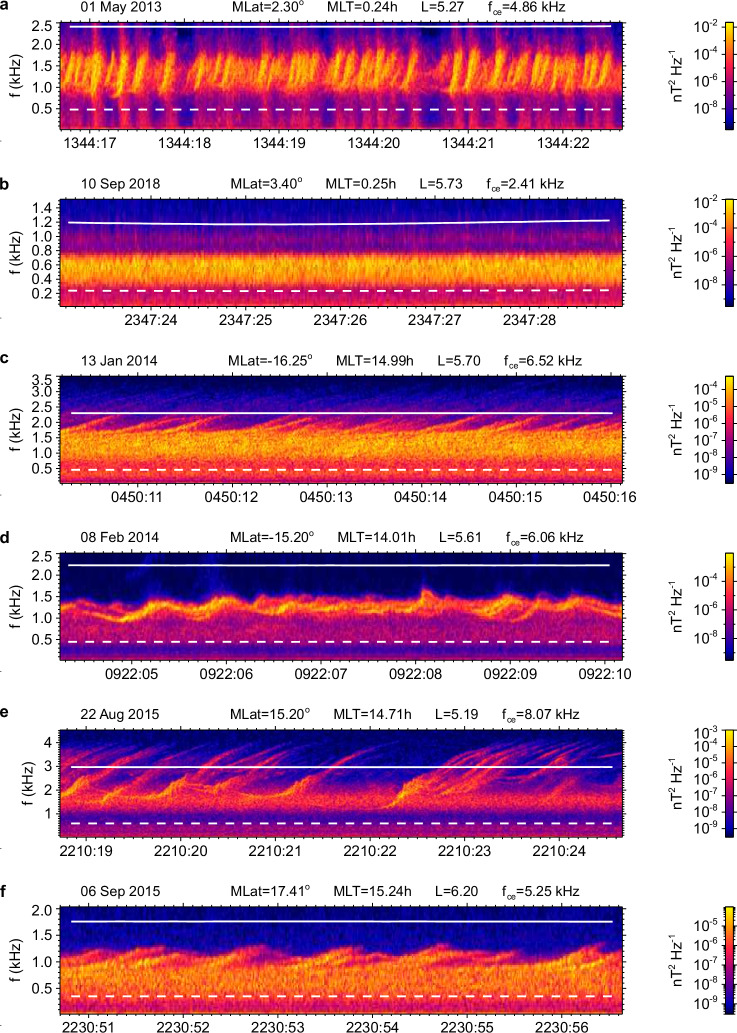
Examples of high-resolution spectrograms. Frequency–time power spectrograms of the trace of the magnetic power spectral density matrix obtained from the continuous burst mode captures by the EMFISIS Waves instrument on Van Allen Probe A. **a**, **b** Examples from the pre-midnight boundary of the occurrence peak in Fig. 2e, within 5° from the geomagnetic equator and at MLT between 22 and midnight. **c–f** Examples from the afternoon-side boundary of the main peak of occurrence in Fig. 2a, at 14–16 MLT, and at latitudes of 15–20° from the geomagnetic equator. In the first 0.468 s of each of these spectrograms, the survey mode amplitudes at analyzed frequencies between the dashed and solid white lines reach **a** 200 pT, **b** 137 pT, **c** 216 pT, **d** 27 pT, **e** 127 pT, and **f** 67 pT, respectively. The corresponding sound files are downloadable as Supplementary Audio 01–06.

High-resolution spacecraft measurements of whistler-mode waves are usually triggered by high amplitude signals^33,35^, and therefore the currently available collections of spacecraft measurements do not allow us to build large unbiased databases separately for chorus and exohiss. It might be possible that some local time—latitude bins in Fig. 2 would predominantly contain exohiss, that chorus would prevail elsewhere, and that their amplitudes would be systematically different. We therefore randomly verified individual cases, which landed in the same bin, and for which we have high-resolution continuous waveform data. For example, within 5° from the equator and with MLT between 22 and midnight we observe a pre-midnight boundary of the pronounced occurrence peak for additional constraints of high wave amplitudes in Fig. 2e, f. Figure 5a, b shows that this bin can contain intense discrete chorus elements, but also intense hiss (Supplementary Audio 1–2). It might seem, though, that only measurements from a very small number of particular time intervals with intense waves fill this bin. A closer inspection of the dataset shows that this is not the case: without additional constraints this bin contains 353 thousand measurements, with 54% occurrence rate, originating from 287 different days; with an additional constraint of waves with effective squared amplitudes above 10^4^ pT^2^, this bin still shows over 22 thousand measurements from 170 different days and an occurrence rate of 3.4%, and the additional constraint of squared amplitudes above 10^5^ pT^2^ results here in 2.6 thousand measurements from 92 different days and an occurrence rate of 0.4%.

For another example, at the dusk-side boundary of the noon-centered main peak of occurrence in Fig. 2a, at MLT between 14 and 18, and at latitudes between 15° and 20° from the geomagnetic equator, we also observe different forms and intensities of both discrete emissions and exohiss (Fig. 5c–f and Supplementary Fig. 8a–f, Supplementary Audio 3–12). This is observed generally in other bins as well. Our examples are therefore in agreement with the statement^15^ that the generation mechanism of the hiss-like band and discrete chorus may have a close relationship. It was based on observations of the THEMIS spacecraft, which showed that polarization properties of exohiss and rising tone chorus were very similar, and exohiss emissions were only slightly weaker than rising tone chorus^15^.

The obtained heavy-tail distributions of chorus/exohiss squared amplitudes affect their long-term average values, which are used in empirical models for quasilinear calculations of diffusion coefficients and lifetimes of radiation belt electrons. Previous results show that the long-term average values strongly react to geomagnetic activity levels^24,41^ and that geomagnetic indices can be therefore used to parametrize the empirical models. We show here that the entire distribution shifts to higher amplitudes with increasing activity (Fig. 1j) and the long-term average values are driven by the tail of the distribution (Fig. 1d). Our results imply that the power-law distribution of time intervals between occurrences of squared amplitudes above 10^4^ pT^2^ corresponds to an average value of only 30 min across our entire dataset, with significant clustering of the neighboring detections (Fig. 3d). The occurrence rate of these waves peaks at 4% in the low-latitude dawn sector (Fig. 2e). The occurrence rate of waves with 10 times larger squared amplitudes peaks in the same region at 0.4% (Fig. 2f).

Therefore, these very intense waves contribute equally to the long-term average of the squared amplitudes in this region, but the power-law exponent of distribution of time intervals between their detections is lower, resulting in an average value close to 10 h across our entire dataset (Fig. 3e). This may be comparable to the time scales of quasilinear diffusion, which typically are hours to days^2–4^. Such intense lower-band waves, therefore, may not occur in our data set during particular intervals at the diffusion time scales, but they still strongly influence the wave models, which are based on long-term average values accumulated during the spacecraft operational period of many years. Intense waves at particular frequencies within the lower band may be absent for even longer periods.

It is important to note here that our data set^37^ consists of wave power averaged during approximately 468-ms intervals for Van Allen Probes and 3.84-s intervals for Cluster, while actual chorus wave packets mainly consist of trains of relatively short (typically 5–100 ms) and intense subpackets^21,62,66–68^. The tail of the distribution of the squared instantaneous amplitudes of chorus^66,68^ then would probably extend to larger values compared to the distributions from Fig. 1, while the wave packet variability, including wave phase and amplitude variability, may still allow quasi-linear theory to remain applicable^69–71^.

More detailed studies are needed to compare the onboard survey data products with the instantaneous amplitudes and frequencies of the original waveforms and with their averages over the duration of diffusion events. Ensemble modeling with the entire spatio-temporal distribution of amplitudes might then be an appropriate approach to verify the inputs for quasilinear simulations of particle fluxes in the radiation belts.

## Methods

### The dataset

We analyzed a dataset^37^ of root-mean-square amplitudes of right-hand polarized whistler mode chorus and exohiss with signed ellipticities^72^ above 0.2, in a lower band frequency range between 0.1 *f*_ce0_ and 0.5 *f*_ce0_, where *f*_ce0_ is the equatorial electron cyclotron frequency. In the approximation of the dipole magnetic field lines, we obtain it as

4 $${f}_{{{\rm{ce}}}0}={f}_{{{\rm{ce}}}}{\cos }^{6}{{\rm{MLat}}}/\sqrt{1+3{\sin }^{2}{{\rm{MLat}}}},$$

where *f*_ce_ is the local electron cyclotron frequency obtained from measurement of the background magnetic field at the magnetic latitude MLat.

The data set is based on the Survey data of the EMFISIS Waves instruments^33,38^ with nearly continuous time coverage from the entire operational period of almost 7 years of both Van Allen Probes, and on the Normal mode data of the STAFF-SA instruments^34,39^ from all four Cluster spacecraft collected during more than 19 years, with 19% time coverage at radial distances below 11 Earth’s radii. The time resolution is 6 s for Van Allen Probes and 4 s for Cluster. The measurements were collected during the maximum and the declining phase of the Solar cycle 23 and the entire Solar cycle 24.

Compared to the original data set^37^ we additionally excluded six brief intervals from the Cluster mission data and 1 brief interval from Van Allen Probe B data (corresponding to 0.07% of data), where the time information was corrupted. We also selected the data inside the plasmatrough, limited on the inner side by the dipole field line at an equatorial distance of 1 Earth’s radius (*R*_E_) outward from the model plasmapause^40^. This offset minimizes possible contamination of our data set by plasmaspheric hiss or lightning whistlers, taking into account the root-mean-square error of the plasmapause model of 0.74 *R*_E_^40^. At the outer boundary, our analysis is limited by the field line at equatorial distance of 6 *R*_E_ from the model plasmapause and by the model magnetopause^73^. This additional selection procedure resulted in the total number of 5.2 × 10^7^ data captures.

A recently published method^37^ based on a scaled ${\chi }^{2}$ distribution of instrumental noise also allowed us to characterize frequency-dependent detection thresholds and their evolution during the lifetime of the two spacecraft missions, with a low probability of 10^−6^ for a false positive detection throughout our data set^37^. This procedure resulted in 2.7 × 10^7^ chorus/exohiss detections, corresponding to an overall occurrence probability of 52%.

### Discretization of the parametric space

We determine the wave parameters using discrete bins in the 4D parameter space. The position within the magnetosphere is given by the magnetic local time (MLT), the absolute value of the magnetic latitude |MLat| related to the Earth’s main dipole axis, and by the *L* parameter calculated from the spacecraft position using the dipole approximation,

5 $$L=R{\cos }^{-2}{{\rm{MLat}}},$$

where *R* is the radial distance from the Earth’s center in Earth radii (*R*_E_, defined as 6371.2 km).

We use 12 bins for MLT: 0 ≤ MLT < 2 h, 2 h ≤ MLT < 4 h, …, 22 h ≤ MLT < 24 h. |MLat| is divided into 13 bins: 0 ≤ |MLat | <5°, 5° ≤ |MLat | <10°,…, 55° ≤ |MLat | <60°, and |MLat | ≥ 60°. We also use 13 bins in *L*: 2 ≤ *L* < 2.5, 2.5 ≤ *L* < 3, …, 7.5 ≤ *L* ≤ 8, and *L* > 8 (Supplementary Fig. 1a-c) or in *L* − *L*_PP_: 0 ≤ *L* − *L*_PP_ < 0.5, 0.5 ≤ *L* − *L*_PP_ < 1, …, 5.5 ≤ *L* − *L*_PP_ ≤ 6, and *L* − *L*_PP_ > 6 (Fig. 1g-i).

Besides these positional parameters, external drivers of the waves are characterized by the planetary *K*_P_ index in 10 bins: 0 and 0 + , 1*–* and 1, 1+ and 2*–*,…. 5+ and 6*–*, 6 and above (Fig. 1j–l) or by the −AL* index obtained as the negative minimum of the auroral AL index over the last 3 h in 8 logarithmically spaced bins between 10 nT and 1000 nT, and by 2 additional bins, −AL* <10 nT and −AL* > 1000 nT, resulting in 10 − AL* bins (Supplementary Fig. 1d–f). The total number of all bins in the 4D parameter space is 20280. It is often practical to reduce the number of bins by joining them into a larger set of adjacent bins along selected dimensions of the parameter space. For example, in Fig. 2a we use all 12 MLT bins and all 13 |MLat| bins but we select 10 bins in *L*−*L*_PP_ from 1 to 6 and join them together with all 10 *K*_P_ bins.

### Characteristics of the distributions of squared amplitudes

The occurrence rate

6 $${r}_{b}={M}_{b}/{N}_{b},$$

is defined in a bin b of the parameter space by the ratio of the number *M*_*b*_ of observations with ellipticity *E* above 0.2, and with the magnetic power spectral density above the noise threshold (defined by the probability of 10^−7^) in at least one frequency channel contained inside a predefined frequency interval, and the total number of observations *N*_*b*_ in a given bin. The predefined frequency interval is linked to the equatorial electron cyclotron frequency *f*_ce0_, corresponding to the lower band chorus in the frequency range between 0.1 *f*_ce0_ and 0.5 *f*_ce0_. The occurrence percentage then reads

7 $${R}_{b}={r}_{b} 100 \%=\frac{{M}_{b}}{{N}_{b}} 100 \%$$

The distribution of squared amplitudes in a bin *b* is estimated by a histogram *H*_*bi*_, *i* = 1…26, which is constructed out of *M*_*b*_ observations. The squared amplitude for each observation is obtained from all frequency channels contained in the predefined frequency interval, for which the ellipticity is above 0.2 and magnetic power spectral density is above the *P*_0_ = 10^−7^ noise threshold. The resulting squared amplitudes are accumulated in 24 logarithmically spaced histogram bins between 10^−2^ and 10^6^ pT^2^ and in the additional 2 histogram bins for squared amplitudes below 10^−2^ pT^2^ and above 10^6^ pT^2^. Hence, the total number of histogram bins is 26, and

8 $${\sum }_{i=1}^{26}{H}_{{bi}}={M}_{b}$$

From these histograms we obtain *p*-percentiles *Q*_*bp*_ to characterize the distribution of squared amplitudes in each bin *b* of the parameter space:

9 $${Q}_{{bp}}={S}_{{bj}}+({S}_{b\left(j+1\right)}-{S}_{{bj}})\left(\frac{p}{100\%}-{K}_{{bj}}\right)/({K}_{b\left(j+1\right)}-{K}_{{bj}}),$$

where

10 $${K}_{{bk}}={\sum }_{i=1}^{k}{H}_{{bi}}\frac{1}{{M}_{b}}$$

is an estimate of the cumulative probability distribution, *S*_*bj*_ and *S*_*b*(*j*+1)_ are the squared amplitudes at the boundaries of the *j*th histogram bin, and index *j* is defined such that

11 $${K}_{{bj}}\le \frac{p}{100\%} < {K}_{b\left(j+1\right)}$$

In Fig. 1, we characterize the distribution of squared amplitudes by the *p* = 50% percentile (median value), the *p* = 15.9% and 84.1% percentiles, which would respectively correspond to one standard deviation below and above the mean value of a normal distribution, and the *p* = 2.3% and 97.7% percentiles corresponding to two standard deviations below and above the mean value, respectively.

Histograms *H*_*bi*_ are also used to estimate the mean value *B*_*b*_ of the distribution of squared amplitudes in each bin *b* of the parameter space:

12 $${B}_{b}=\frac{1}{{M}_{b}}{\sum }_{i=1}^{26\,}{H}_{{bi}}\sqrt{{S}_{b\left(i+1\right)}{S}_{{bi}}}$$

where geometrical averages of the bin boundaries are used to account for their logarithmic spacing.

### Significance of results obtained from a binned data set

The coverage of different MLT sectors by our data set is nearly uniform (see Supplementary Fig. 7a), while we couldn’t avoid large differences in coverage of different geomagnetic latitudes. Van Allen Probes contribute by more than one-third of the data set and their orbit is confined within 20° from the geomagnetic equator, while the Cluster data set is unevenly distributed to all latitudes, with a decreasing number of measurements toward high latitudes. As a consequence, the number of measurements per 5° latitude bin is by one order of magnitude larger below 20° than at latitudes around 40° and by two orders of magnitude larger compared to latitudes above 50°. This does not represent a major flaw in our analysis as long as the number of measurements at high latitudes can still ensure a reasonable statistical significance of our results. The random error of the occurrence percentage *R*_*b*_ can be estimated as

13 $$\sigma \left({R}_{b}\right)=\frac{{R}_{b}}{\sqrt{{M}_{b}}}\left(\frac{1}{\sqrt{{R}_{b}/100 \% }}+1\right),$$

assuming that the individual measurements are independent. At latitudes above 50°, our coverage on the order of 10^4^ measurements per bin (Supplementary Fig. 7a) is therefore sufficient for a reasonable absolute precision of 0.4% to 1.7% for the observed occurrence percentages between 10% and 80% (Fig. 3a).

Similarly, a separate analysis of the inner and outer plasmatrough region (Supplementary Figs. 2 and 3) is also based on a reasonable number of accumulated measurements (Supplementary Fig. 7b and 7c), although the outer region suffers from the absence of Van Allen Probes measurements at low latitudes. For highly constrained selections of data (for example, Supplementary Fig. 3f), the precision rapidly decreases, as an additional selection of data results in some regions of the parametric space not being adequately populated by the measurements. Selecting observations for separate phases of the Solar cycle also led to very low coverage at high latitudes (above 55° in Supplementary Fig. 7d) and hence to large random fluctuations of the results at these latitudes (Supplementary Fig. 4c).

An important aspect of this problem is the assumption of independence of measurements. The measurements come from two spacecraft constellations, within each of which the separation distances between the measurement locations varied in time. If the spacecraft were closer than the characteristic spatial dimensions of the observed wave emissions, their measurements would be strongly dependent on each other. For Cluster measurements, the distances between separate pairs of spacecraft seldom decreased below 100 km^74^ which would be comparable to characteristic perpendicular scales of discrete chorus elements^61–64^, but somewhat more often they decreased below 1000 km, which would be relevant to observations of the less frequently observed obliquely propagating chorus^17,75^. Van Allen Probes also periodically approached these smaller inter-spacecraft separation distances but only for a small fraction of time compared to the duration of the mission.

More importantly, the cadence of one measurement per 4–6 s is quite high, and this is probably the main source of violations of the assumption of independence of measurements on each spacecraft. Taking into account the typical spacecraft orbital speed in the analyzed region and the above-mentioned characteristic spatial scales of 100–1000 km, they would be traversed in 25–250 s, which may lead to a reduction of the number of independent measurements by a factor of 4–60. This factor also roughly corresponds to reasonable scales of temporal variations of occurrence patterns on the order of minutes (corresponding to 10–100 measurements). The corresponding correction of *M*_*b*_ leads, according to Eq. (13), to an increase of estimated uncertainties by a factor of 2–8, which is still sufficient to ensure a good statistical significance of our main results.

### Probability density function of time delays between detections

The time delays were calculated after combining the entire set of lower band chorus/exohiss measurements from both Van Allen Probes and all four Cluster spacecraft into a single sequence ordered by the time of each separate measurement *t*_*i*_. The plasmatrough measurements with *L*−*L*_PP_ between 1 and 6 were selected. If an additional amplitude threshold was used, a corresponding subset of *n* measurements from this sequence of all chorus/exohiss detections was analyzed. Time delays *d*_*i*_ = *t*_*i*+1_− *t*_*i*_ (where *i* = 0…*n* − 1) between neighboring detections from this subset, served us to construct a histogram of counts *H*_*j*_ in 50 logarithmically distributed bins (*j* = 0…49) between *D*_0_ = 10 s and *D*_50_ = 4.5 h, where the boundaries between bins were defined as

14 $$\log {D}_{j}=\log {D}_{0}+\frac{j}{49}\left(\log {D}_{50}-\log {D}_{0}\right)$$

The probability density function was then estimated in each bin,

15 $${{{\rm{PDF}}}}_{j}=\frac{{H}_{j}}{n\left({D}_{j+1}-{D}_{j}\right)}$$

## Supplementary information

Supplementary Information

Description of Additional Supplementary Files

Supplementary Dataset 1

Supplementary Dataset 2

Supplementary Dataset 3

Supplementary Dataset 4

Supplementary Dataset 5

Supplementary Dataset 6

Supplementary Dataset 7

Supplementary Dataset 8

Supplementary Dataset 9

Supplementary Dataset 10

Supplementary Dataset 11

Supplementary Dataset 12

Transparent Peer Review file

## Source data

Source Data

## Data Availability

The Van Allen Probes EMFISIS data set is available from the University of Iowa archive on https://emfisis.physics.uiowa.edu. The Cluster STAFF-SA data set is available from the ESA Cluster Science Archive on https://csa.esac.esa.int. The *K*_P_ and AL indices of geomagnetic activity are available from the World Data Center for Geomagnetism Kyoto on https://wdc.kugi.kyoto-u.ac.jp/kp/. The data set of integrated lower band chorus/exohiss amplitudes used in this study, together with the data set of detection thresholds, software to reuse the detection thresholds, and a detailed description of the methods to determine the instrumental noise distributions are linked to 10.1038/s41597-025-05531-6^37^. The source data for Figs. 1–4 are provided with this paper in the Source Data file. The source data for Fig. 5 is provided with this paper as Supplementary Audio 01–06. Source data are provided with this paper.

## Code availability

Analysis in this paper was done using the Interactive Data Language software available from NV5 Geospatial Co. Custom code to handle the tables of detection thresholds from 10.1038/s41597-025-05531-6^37^ is available on 10.6084/m9.figshare.29433593. Custom code to read the tables of root-mean-square amplitudes of lower-band whistler mode waves from 10.1038/s41597-025-05531-6^37^ is available on 10.6084/m9.figshare.32866523
