# Supplementary material for: Spatiotemporal patterns of lower-band whistler mode waves in the magnetosphere of Earth
Source: Nat Commun. 2026 Jul 30;17:7569. doi: 10.1038/s41467-026-75552-1 (PMC13424088; doi:10.1038/s41467-026-75552-1)
Supplement: Supplementary file 1 — Supplementary Information [file 41467_2026_75552_MOESM1_ESM.pdf]

Supplementary Information

Spatiotemporal patterns of lower-band whistler mode waves in the magnetosphere of Earth

O. Santolík[1,2,*], I. Kolmašová[1,2], U. Taubenschuss[1], M. Hanzelka[1]

[1] Department of Space Physics, Institute of Atmospheric Physics of the Czech Academy of Sciences, Prague, Czechia

[2] Faculty of Mathematics and Physics, Charles University, Prague, Czechia

*Corresponding author (os@ufa.cas.cz)

Supplementary Figure 1. Distribution of squared amplitudes of lower band chorus/exohiss as a function of the *L* parameter and the auroral activity index AL*

Supplementary Figure 2. Occurrence probabilities of lower band chorus/exohiss close to the plasmapause.

Supplementary Figure 3. Occurrence probabilities of lower band chorus/exohiss for the outer plasmatrough region.

Supplementary Figure 4. Occurrence probabilities of lower band chorus/exohiss for different phases of the Solar cycles 23 and 24.

Supplementary Figure 5. Occurrence probabilities of lower band chorus/exohiss above 100 pT for different phases of the Solar cycles 23 and 24.

Supplementary Figure 6. Occurrence probabilities of lower band chorus/exohiss for different levels of geomagnetic activity.

Supplementary Figure 7. Number of observations in the analyzed data set

Supplementary Figure 8. Examples of high-resolution spectrograms on the dusk side.

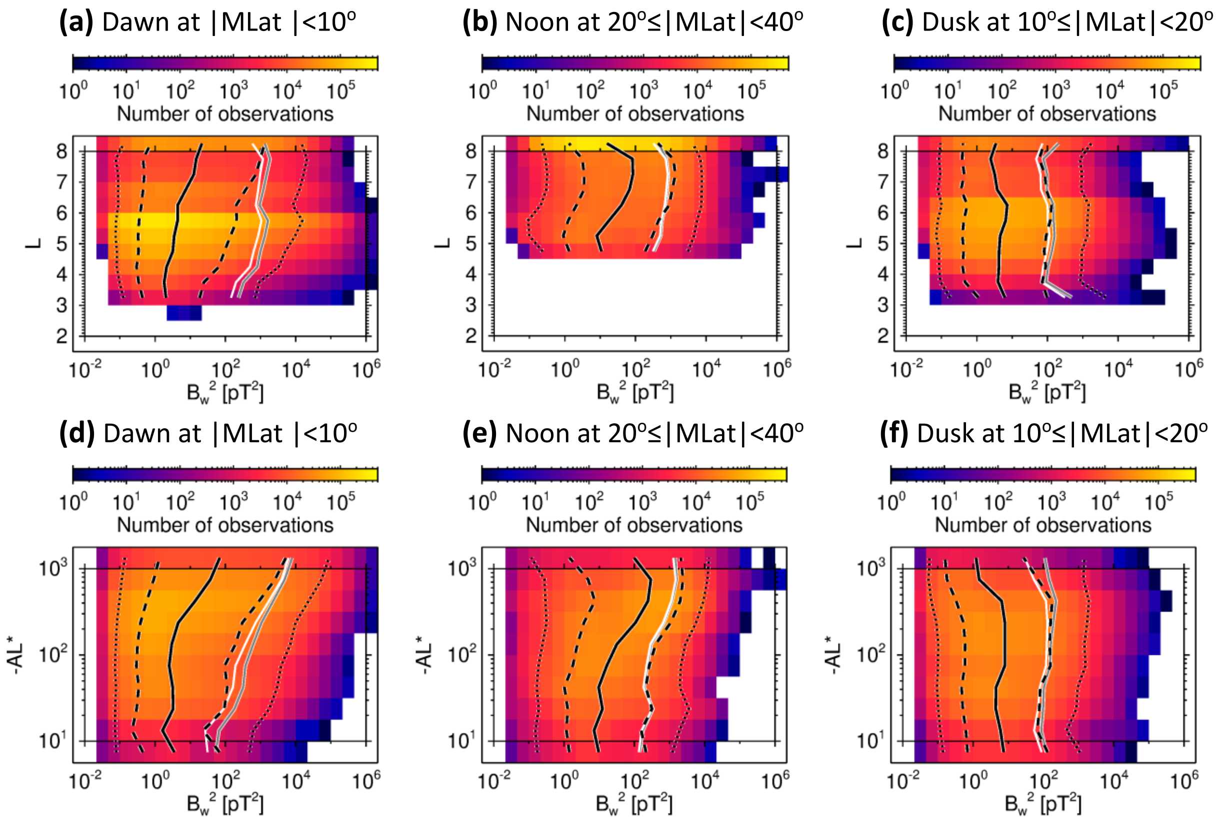

Supplementary Figure 1. Distribution of squared amplitudes of lower band chorus/exohiss as a function of the *L* parameter and the auroral activity index AL*. Set of histograms of the frequency integrated trace of the magnetic power spectral density matrix B_w^2 in **(a–c)** 13 discrete bins of the *L* parameter for three intervals of MLT and |MLat|: **(a)** 0–8 MLT at |MLat | < 10°, **(b)** 10–14 MLT at 20° ≤ |MLat| < 40°, **(c)** 16–20 MLT at 10° ≤ |MLat| < 20°. **(d–f)** in 10 discrete bins of −AL*, obtained as the negative minimum of the auroral activity index AL over the past three hours, for the same intervals of MLT and |MLat|, as in panels a–c. The format is the same as in Figure 1: the median value of B_w^2 is over-plotted by a black solid line, 15.9 and 84.1 percentiles (±1 standard deviation from the median for a normal distribution) by dashed lines, 2.3 and 97.7 percentiles (±2 standard deviations from the median for a normal distribution) by dotted lines; the estimated mean value of B_w^2 is given by a grey solid line, the white solid line shows the long-term average (mean value normalized by the occurrence rate). Source data are provided with this paper.

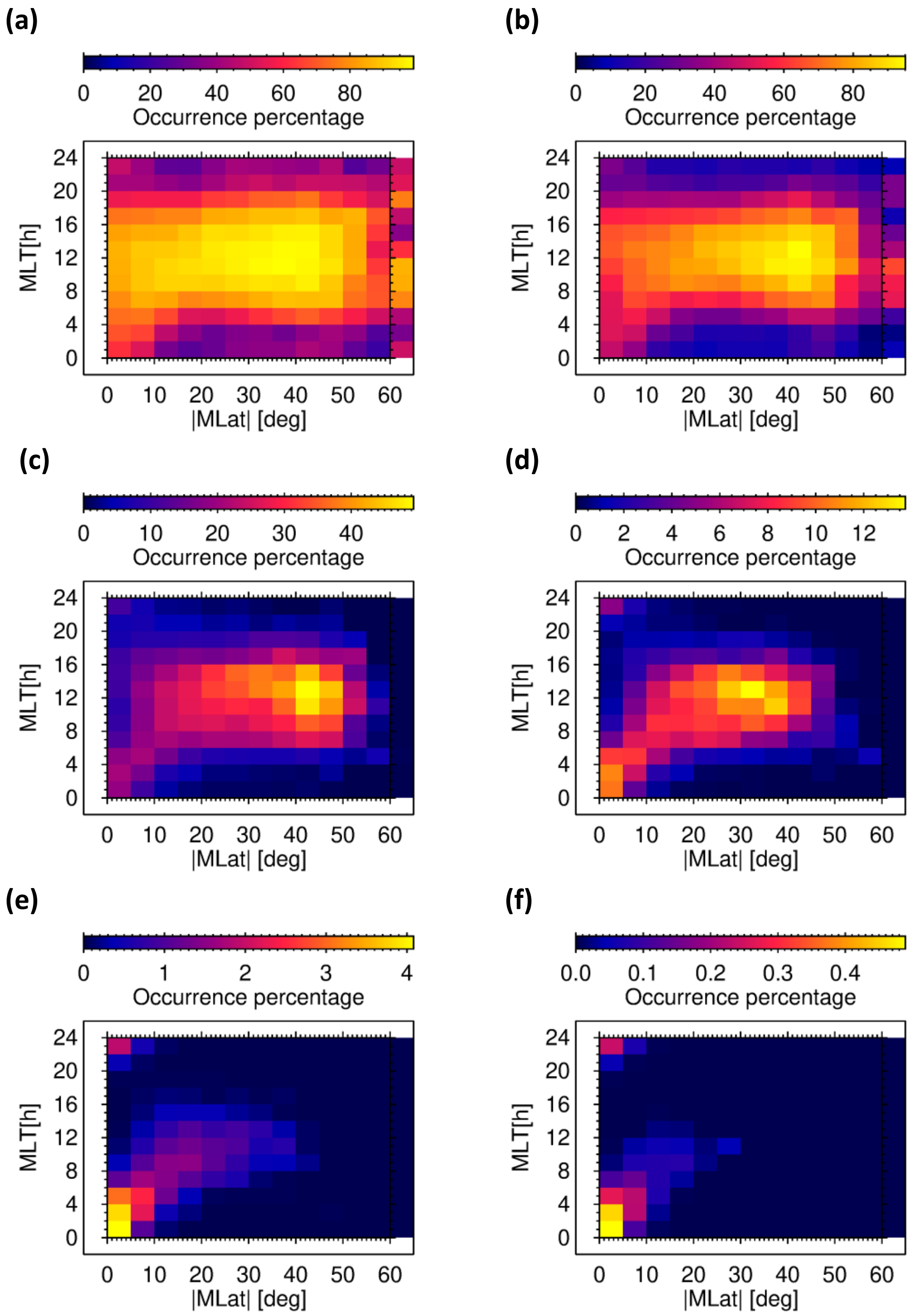

Supplementary Figure 2. Occurrence probabilities of lower band chorus/exohiss close to the plasmapause. The format is the same as in Figure 2 but the data set is restricted to measurements close to the model plasmapause [40] ($1 < L{-}L_{PP} \leq 3$ in the dipole approximation) and within the model magnetopause [73]. **(a)** Occurrence percentage; **(b)** Occurrence percentage with an additional constraint for the trace of the magnetic power spectral density matrix larger than 1 pT^2. The same for the constraint of **(c)** 10^2 pT^2, **(d)** 10^3 pT^2, **(e)** 10^4 pT^2, and **(f)** 10^5 pT^2. Summary results for latitudes above 60° are plotted on the right-hand side of each plot. Source data are provided with this paper.

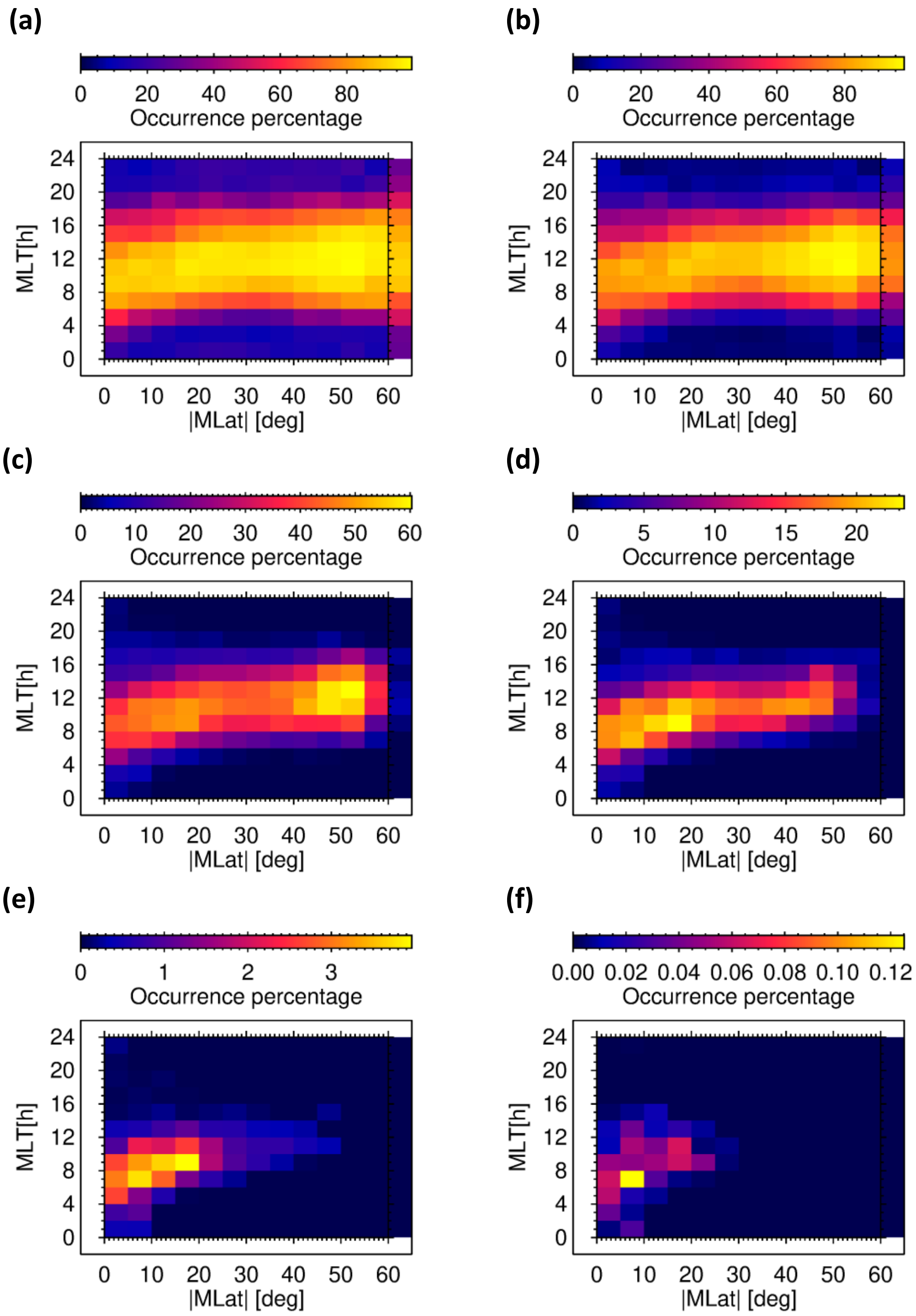

Supplementary Figure 3. Occurrence probabilities of lower band chorus/exohiss for the outer plasmatrough region. The format is the same as in Figure 2 but restricted only to measurements with the equatorial distance from the model plasmapause [40] between 3 and 6 Earth's radii and within the model magnetopause [73]. **(a)** Occurrence percentage; **(b)** Occurrence percentage with an additional constraint for the trace of the magnetic power spectral density matrix larger than 1 pT^2. The same for the constraint of **(c)** 10^2 pT^2, **(d)** 10^3 pT^2, **(e)** 10^4 pT^2, and **(f)** 10^5 pT^2. Summary results for latitudes above 60° are plotted on the right-hand side of each plot. Source data are provided with this paper.

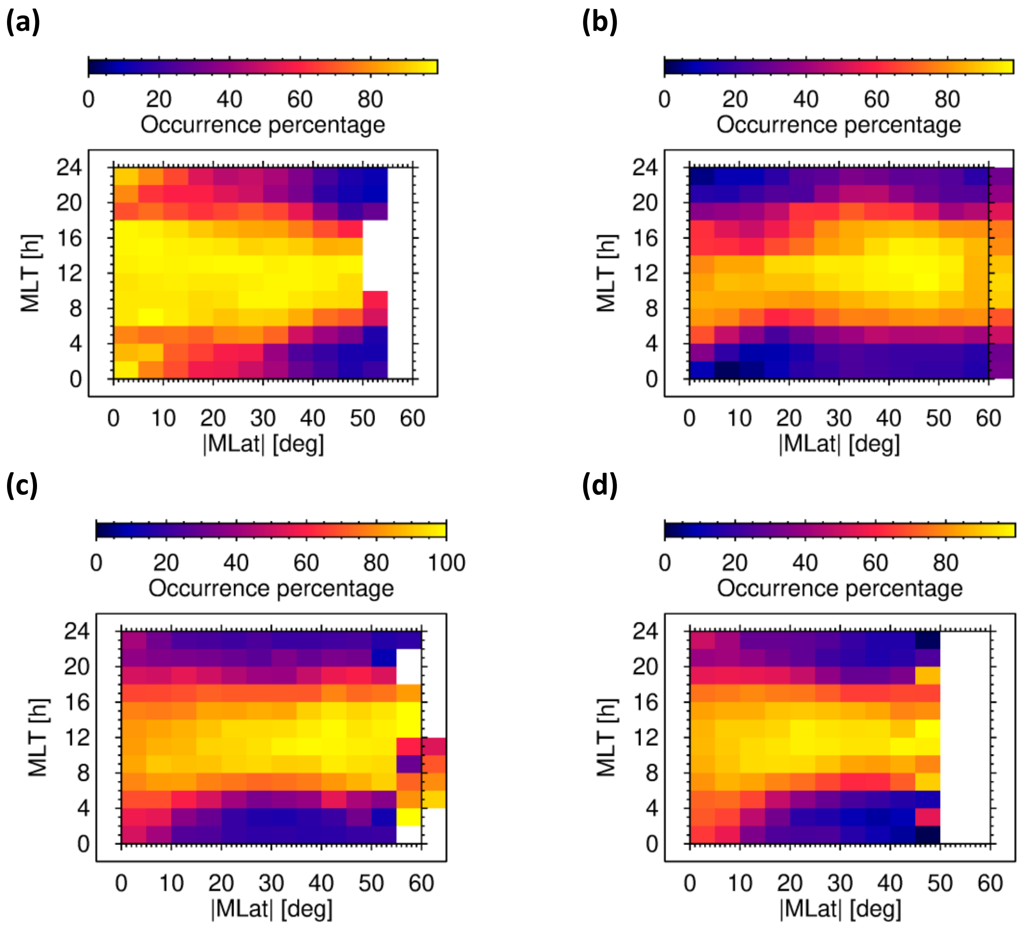

Supplementary Figure 4. Occurrence probabilities of lower band chorus/exohiss for different phases of the Solar cycles 23 and 24. The format is similar as in Figure 2a, for plasmatrough measurements with the equatorial distance from the model plasmapause [40] between 1 and 6 Earth's radii and within the model magnetopause [73]. **(a)** Measurements from 7 January 2001 till 5 November 2005, during the maximum and declining phase of Solar cycle 23. **(b)** Measurements from 5 November 2005 till 4 September 2010, around the minimum of solar activity. **(c)** Measurements from 4 September 2010 till 3 July 2015, during the rising phase and maximum of Solar cycle 24. **(d)** Measurements from 3 July 2015 till 30 April 2020, during the declining phase of Solar cycle 24 and the minimum of solar activity after it. Source data are provided with this paper.

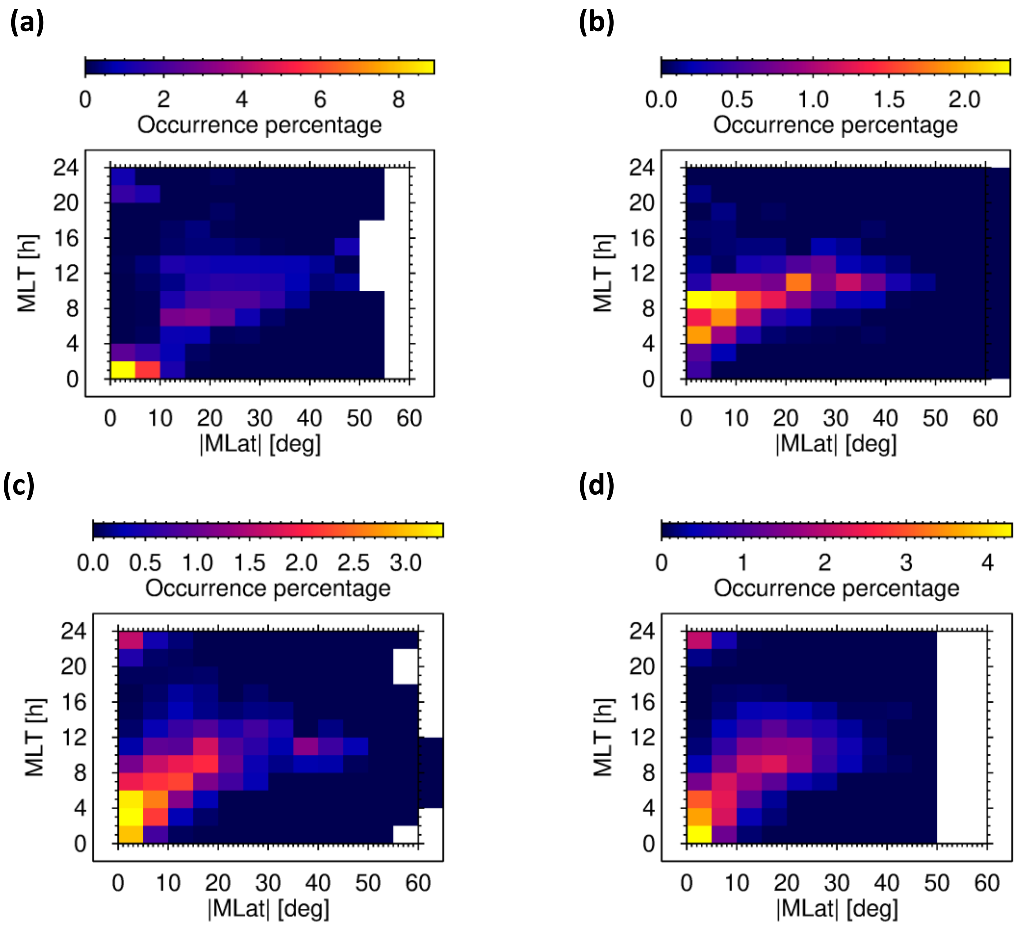

Supplementary Figure 5. Occurrence probabilities of lower band chorus/exohiss above 100 pT for different phases of the Solar cycles 23 and 24. The format is similar as in Figure 2e, for plasmatrough measurements with the equatorial distance from the model plasmapause [40] between 1 and 6 Earth's radii and within the model magnetopause [73], with an additional constraint for the trace of the magnetic power spectral density matrix larger than 10^4 pT^2 **(a)** Measurements from 7 January 2001 till 5 November 2005, during the maximum and declining phase of Solar cycle 23. **(b)** Measurements from 5 November 2005 till 4 September 2010, around the minimum of solar activity. **(c)** Measurements from 4 September 2010 till 3 July 2015, during the rising phase and maximum of Solar cycle 24. **(d)** Measurements from 3 July 2015 till 30 April 2020, during the declining phase of Solar cycle 24 and the minimum of solar activity after it. Source data are provided with this paper.

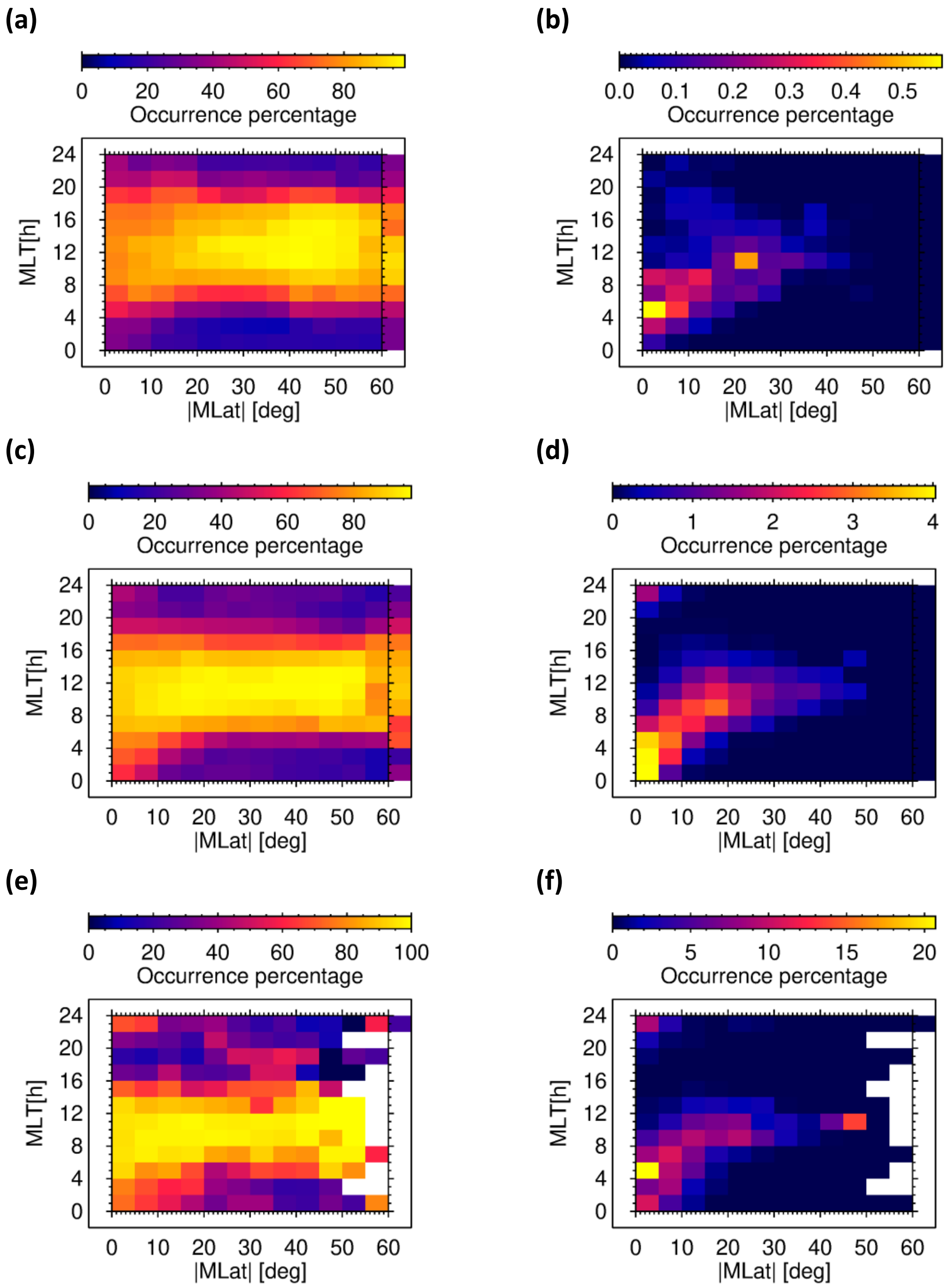

Supplementary Figure 6. Occurrence probabilities of lower band chorus/exohiss for different levels of geomagnetic activity. The format is similar as in Figure 2 but for **(a)** selected measurements during low geomagnetic activity with $-AL^* \leq 100$ nT in 38% of our data set, where $-AL^*$ is the negative minimum of the auroral AL index over the past 180 1-minute averages; **(b)** the same with an additional constraint for the trace of the magnetic power spectral density matrix larger than 10^4 pT^2; **(c, d)** the same as (a, b), respectively, but for selected data during moderate activity, with 100 nT $< -AL^* \leq$ 1000 nT in 60% of our data set; **(e, f)** the same as (a, b), respectively, but for the selected data set during high activity, with $-AL^* >$ 1000 nT in only 2% of our original data set. Source data are provided.

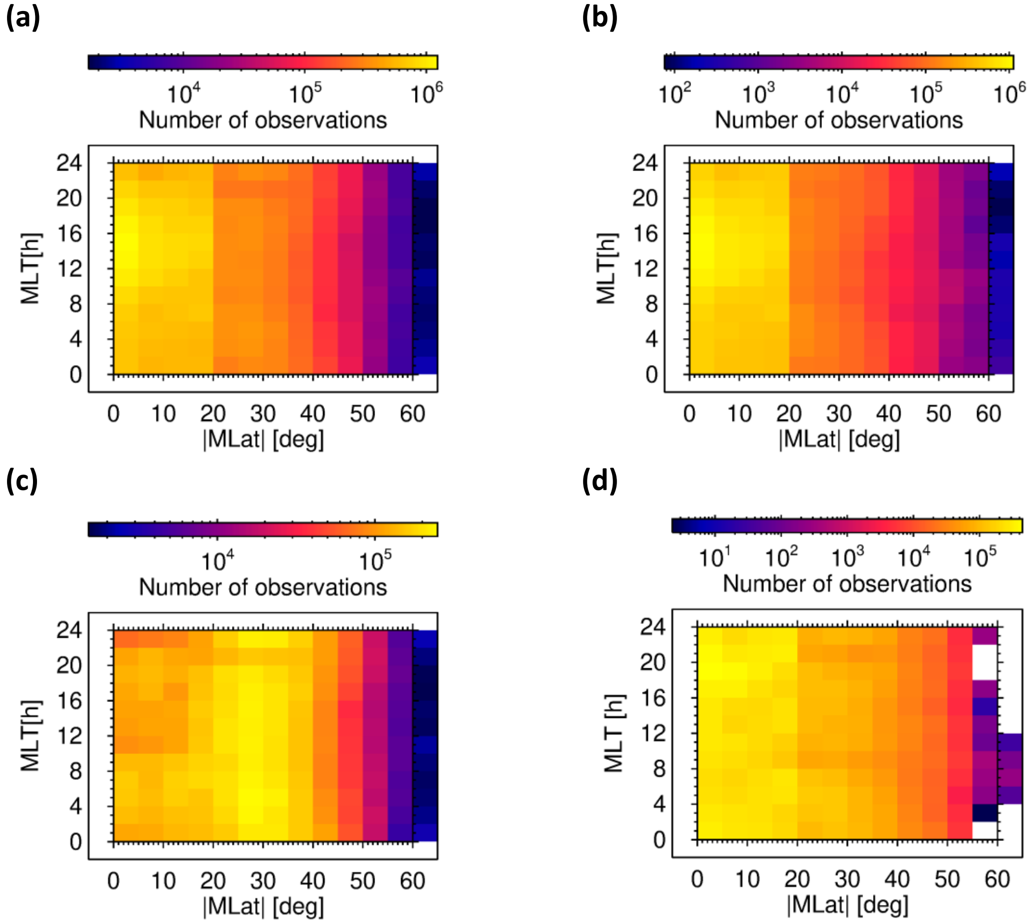

Supplementary Figure 7. Number of observations in the analyzed data set. A joint data set of two Van Allen Probes and four Cluster spacecraft is accumulated in 12×13 discrete bins in magnetic local time MLT and absolute value of the magnetic latitude |MLat| up to 60°, for the equatorial distance from the plasmapause [40] ($L - L_{PP}$) between 1 and 6 Earth's radii and within the magnetopause [73]. **(a)** Distribution of all observations in the data set, relevant to Figure 2. **(b)** Distribution of selected observations close to the model plasmapause for $1 < L - L_{PP} \leq 3$ in the dipole approximation, relevant to Supplementary Figure 2a. **(c)** Distribution of selected observations in the outer region of the plasmatrough for $3 < L - L_{PP} \leq 6$ in the dipole approximation and within the model magnetopause, relevant to Supplementary Figure 3.
(d) Example of the distribution of selected observations in a limited time period from 4 September 2010 till 3 July 2015 during the rising phase and maximum of Solar cycle 24, relevant to Supplementary Figures 4c and 5c. Source data are provided with this paper.

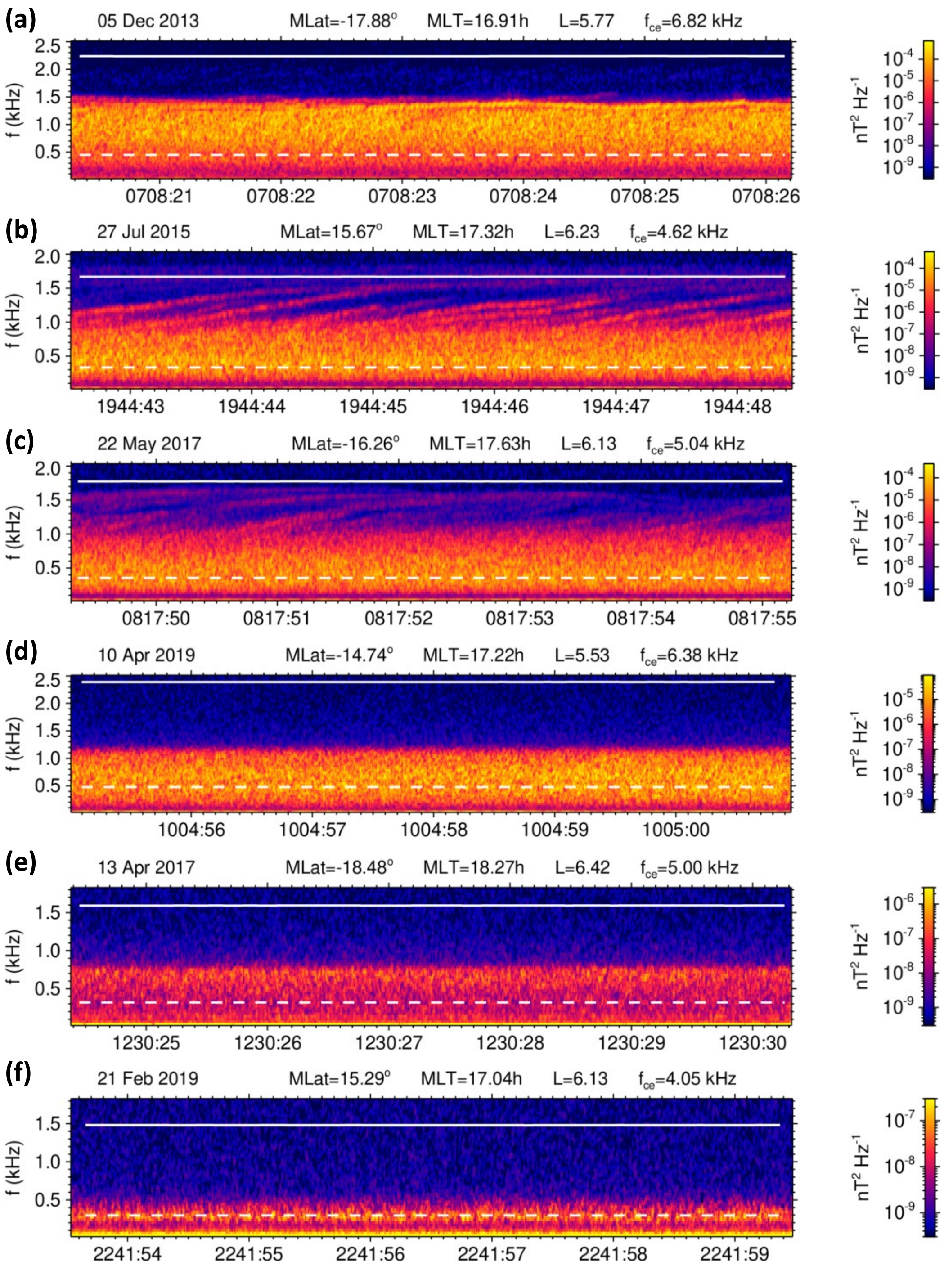

Supplementary Figure 8. Examples of high-resolution spectrograms on the dusk side. Frequency-time power spectrograms of the trace of the magnetic power spectral density matrix obtained from continuous burst mode waveform captures by the EMFISIS Waves instrument on Van Allen Probe A. Examples from the dusk-side boundary of the main peak of occurrence in Figure 2a, at MLT of 16:00–18:00, and at latitudes of 15º–20º from the geomagnetic equator. In the first 0.468 s of each of these spectrograms, the survey mode amplitudes in the analyzed frequency interval between the dashed and solid white lines reach **(a)** 200 pT, **(b)** 137 pT, **(c)** 86 pT, **(d)** 63 pT, **(e)** 6.1 pT, and **(f)** 1.8 pT, respectively. The corresponding sound files are downloadable as Supplementary Audio 07-12.
