# Supplementary material for: Spatiotemporal patterns of lower-band whistler mode waves in the magnetosphere of Earth
Source: Nat Commun. 2026 Jul 30;17:7569. doi: 10.1038/s41467-026-75552-1 (PMC13424088; doi:10.1038/s41467-026-75552-1)
Supplement: Supplementary file 2 — Description of Additional Supplementary Files [file 41467_2026_75552_MOESM2_ESM.pdf]

Description of Additional Supplementary Files

1. Supplementary Audio 01. Audio file corresponding to Figure 5a. The waveforms, from which the spectrogram was obtained were transformed into a stereo wave file using two magnetic field components perpendicular to the local magnetic field line.
2. Supplementary Audio 02. Audio file corresponding to Figure 5b. The waveforms, from which the spectrogram was obtained were transformed into a stereo wave file using two magnetic field components perpendicular to the local magnetic field line.
3. Supplementary Audio 03. Audio file corresponding to Figure 5c. The waveforms, from which the spectrogram was obtained were transformed into a stereo wave file using two magnetic field components perpendicular to the local magnetic field line.
4. Supplementary Audio 04. Audio file corresponding to Figure 5d. The waveforms, from which the spectrogram was obtained were transformed into a stereo wave file using two magnetic field components perpendicular to the local magnetic field line.
5. Supplementary Audio 05. Audio file corresponding to Figure 5e. The waveforms, from which the spectrogram was obtained were transformed into a stereo wave file using two magnetic field components perpendicular to the local magnetic field line.
6. Supplementary Audio 06. Audio file corresponding to Figure 5f. The waveforms, from which the spectrogram was obtained were transformed into a stereo wave file using two magnetic field components perpendicular to the local magnetic field line.
7. Supplementary Audio 07. Audio file corresponding to Supplementary Figure 8a. The waveforms, from which the spectrogram was obtained were transformed into a stereo wave file using two magnetic field components perpendicular to the local magnetic field line.
8. Supplementary Audio 08. Audio file corresponding to Supplementary Figure 8b. The waveforms, from which the spectrogram was obtained were transformed into a stereo wave file using two magnetic field components perpendicular to the local magnetic field line.
9. Supplementary Audio 09. Audio file corresponding to Supplementary Figure 8c. The waveforms, from which the spectrogram was obtained were transformed into a stereo wave file using two magnetic field components perpendicular to the local magnetic field line.
10. Supplementary Audio 10. Audio file corresponding to Supplementary Figure 8d. The waveforms, from which the spectrogram was obtained were transformed into

a stereo wave file using two magnetic field components perpendicular to the local magnetic field line.

11. Supplementary Audio 11. Audio file corresponding to Supplementary Figure 8e. The waveforms, from which the spectrogram was obtained were transformed into a stereo wave file using two magnetic field components perpendicular to the local magnetic field line.
12. Supplementary Audio 12. Audio file corresponding to Supplementary Figure 8f. The waveforms, from which the spectrogram was obtained were transformed into a stereo wave file using two magnetic field components perpendicular to the local magnetic field line.
