# Supplementary material for: Spatiotemporal patterns of lower-band whistler mode waves in the magnetosphere of Earth
Source: Nat Commun. 2026 Jul 30;17:7569. doi: 10.1038/s41467-026-75552-1 (PMC13424088; doi:10.1038/s41467-026-75552-1)
Supplement: Supplementary file 15 — Transparent Peer Review file [file 41467_2026_75552_MOESM15_ESM.pdf]

nature portfolio

Peer Review File

patiotemporal patterns of lower-band whistler mode waves in the magnetosphere of Earth

Corresponding Author: Professor Ondrej Santolik

This file contains all reviewer reports in order by version, followed by all author rebuttals in order by version.

Version 0:

Reviewer comments:

Reviewer #1

(Remarks to the Author)
Review of “Prevailing dayside occurrence of lower band whistler mode chorus and exohiss” by Santolik, Kolmasova, Taubenschuss and Hanzelka
The paper is very well written, contains important new information and should be published in Nature Communicates after some minor corrections have been made.

Comments
Abstract lines 12 and 13: The use of 4 Cluster spacecraft data and two Van Allen Probes data is a case of oversampling since the 4 Cluster spacecraft collect essentially same chorus and exohiss data.
Line 17. “The peak of chorus occurrence being located symmetrically about local noon” and Figures 3a and 3b. This distribution is most likely dominated by the lowest intensity waves of your study. Please use high time resolution chorus/hiss data taken at 1700 local time to examine 10 to 20 samples to confirm that these low intensity events are indeed chorus and hiss and report this in the paper. Right now it is uncertain what this symmetry is due to.
Figure 3 is not in contradiction to the prevailing scenario of chorus generation. There are three predominant sources of chorus: one is local midnight injection of anisotropic ~10 to 100 keV substorm electrons (your reference 1). A second source is when the drifting energetic electron cloud crosses the dawn boundary, the enhanced thermal plasma density leads to larger growth rates of chorus and thus the original name “dawn chorus”. The third is solar wind pressure pulses on the dayside magnetosphere (see JGR, 117, A00L12, 2012 doi?10.1029.20011JA017180; JGRSP, 121, 2016 doi:10.1002/2016JA022499). It should be noted that in the year long survey (much shorter than yours), in JGR, 82, 32, 5112-5128, 1977 the midnight to dawn sector chorus was much weaker than the dayside chorus. So these scenarios generally fit with your Figures 1c and 1d.
Lines 17-19. I think this is in agreement with the 1977 paper mentioned above. I will say more about Figure 3e and 3f later.
Lines 29-40. Very nice set of statements. This needs to be told to the readership. Thank you.
Line 37. I suggest adding a reference here. JGRSP, 125, 2020 doi.org/10.1029/2020JA028090.
Lines 61-65. This may be too strong of a statement. I think it is only for the lowest amplitude chorus/exohiss waves.
Figure 3. Please indicate the differences in the 6 panels in the Figure. This will be more convenient for the readership instead of trying to dig it out of the caption.
Lines 130-133. Please say something about Figures 3e and 3f. This very high intensity chorus/exohiss at midnight seems peculiar. I assume that there are very few events here? Perhaps a magnetic storm where the particle injection was extreme, giving extreme electron anisotropies? This high amplitude chorus is at odds with the intensities found in your reference 1. Please take a look using high time resolution data at this event.
Line 275. Please add the reference JGR 114, A03207, 2009 doi:10.1029/2008JA013353 here.
Line 280-281. This is a bit of an overstatement. Only for low intensity chorus and exohiss?
Lines 295-297. I agree. But please look at some of your low intensity events to make sure what they are. Also report it in the paper.

Reviewer #2

(Remarks to the Author)

This manuscript presents a study of the occurrence rate of lower band whistler mode chorus and exohiss waves in the inner magnetosphere of the Earth, based on a combination of well inter-calibrated data from the Cluster four-spacecraft mission and from the Van Allen Probe two-spacecraft mission.
The main results of the manuscript are that:
(a) the overall occurrence rate of chorus waves (of both low and high amplitudes, but mainly low amplitudes) has a large peak at 8-16 MLT, where occurrences are much larger than at 22-6 MLT,
(b) the peak occurrence rate of chorus waves moves from 8-16 MLT to 0-6 MLT at low latitudes as chorus wave amplitude increases from < 30 pT to > 100 pT, but remains at 8-16 MLT at high latitudes.

The problem with this manuscript is that these two main results, (a) and (b), are not new at all, as easily demonstrated below. Exactly the same results have already been published based on THEMIS and Cluster statistics. Therefore, the present manuscript does not contain sufficient new results to be published in Nature Communications.

1. It is found in the present manuscript that (a) the occurrence rate of chorus waves of low amplitudes (<30 pT) has a large peak at 8-16 MLT, where occurrences are much larger than at 22-6 MLT.
Unfortunately, exactly the same result has already been found by Li et al. 2009 (doi:10.1029/2009GL037595) using chorus wave Filter Bank Data statistics from THEMIS spacecraft (it consists of 4-second wave data as in the submitted manuscript). Figure 3(left column) from Li et al. 2009 shows the same peak of occurrences of chorus waves at 8-14 MLT, at both low (<10 degrees, top panel in left column) and high (10-25 degrees, bottom panel in left column) latitudes, for waves of low amplitudes < 30 pT, exactly as in Figures 3(a-d) from the present manuscript.

Unsurprisingly, a similar peak of occurrences near 12 MLT when keeping ALL chorus waves (i.e., mainly low amplitude waves), very similar to Figure 3(a) of the present manuscript, has also been shown in Figure 3(b,f) from Agapitov et al. 2013 (doi:10.1002/jgra.50312), at both low latitudes (<15 degrees) and high latitudes (>15 degrees), based on the same data from Cluster spacecraft used in the present manuscript.

2. It is found in the present manuscript that (b) the peak occurrence rate of chorus waves moves from 8-16 MLT to 0-6 MLT at low latitudes as chorus wave amplitude increases from < 30 pT to > 100 pT, but remains at 8-16 MLT at high latitudes.
Unfortunately, exactly the same results have already been found by Li et al. 2009 using THEMIS data.
As shown in Figure 3(top row, from left to right) from Li et al. 2009, at low latitudes (< 10 degrees) the peak occurrence rate of chorus waves is shifted from 8-16 MLT for wave amplitudes < 30 pT to 0-6 MLT for wave amplitudes > 100 pT, exactly as in Figure 3(c-f) from the present manuscript. At the same time, Figure 3(bottom row, from left to right) from Li et al. 2009 shows that the peak occurrence rate at high latitudes (10-25 degrees) remains at 8-14 MLT as the wave amplitude increases from 10-30 pT to > 100 pT, exactly as in Figure 3(c-f) of the present manuscript.

Reviewer #3

(Remarks to the Author)
The authors have performed an extensive statistical analysis of in-situ chorus and exohiss amplitude observations spanning approximately two solar cycles. The results show that the traditional picture of post-midnight and dawn chorus occurrence is incomplete. Only the very high amplitude (amplitudes larger than 100 pT) chorus waves exhibit higher occurrence in dawn and post-midnight. When accounting for lower amplitudes as well, the distribution of occurrence peaks around local noon.

Although the thresholds used for the wave detection have been carefully considered, some questions remain regarding the sample statistics.

Major comments

1) There is an issue of the sample size in each MLat bin. The RBSP observations are mostly equatorial (<=20 deg), while the Cluster spacecraft span the entire 0 to 60 deg range. Therefore, one can assume that the amount of data in each MLat bin introduces an imbalance. For the same reason, there are many more data points during the RBSP era (especially for low latitudes) than before. As the authors state under “methods”, each Cluster dataset consists of ~2x107 data captures in the 2001-2020 time period, while each RBSP dataset consists of ~3x107 data captures during 2013-2019. This difference may introduce biases in the statistical analysis and the interpretation of the results. Have the authors checked the results using only Cluster during different time-periods, e.g., Solar cycle 23 vs 24 and/or during different solar cycle phases? Complementary figures with the amount of data at each MLat vs MLT bin (similar to figure 3 in the manuscript) would be very helpful and would contribute substantially to the discussion of the results. Finally, the influence of the magnetospheric activity in terms of the Auroral Indices could possibly reveal if these features are driven (or not) by substorm activity. These figures could be included in the supplementary material.

2) Even though these results may significantly affect the outer radiation belt simulations, the authors do not discuss at all the physical mechanisms that lead to the occurrence peak at the local noon sector. The traditional picture of the peak occurrence in the post-midnight and dawn sectors is attributed to the injection of source electrons from the plasma sheet with a high temperature anisotropy. Is it possible that this temperature anisotropy is higher at local noon?.

Minor comments

1) Lines 22-23: It would be quite informative for the reader to mention here the frequency bands that chorus and hiss usually appear.

2) Lines 12, 57 and 70-72: The sentence “90 years” is misleading. The Van Allen Probes mission begun in late 2012 and ended in mid-2019. This is almost 7 years of measurements. The existence of two spacecraft increases the amount of data (which are mentioned in the methods section) as well as the spatio-temporal coverage within the magnetosphere, but that does imply that one may simply multiply the duration of a mission with the number of satellites. Please mention the exact time-span of the observations instead of the “90 years” statement.

3) Line 63: Define lower band chorus.

4) Line 79: …such as the one of the Van Allen Probes with the data from the high inclination orbit of Cluster,….

5) Lines 83-84: The authors state: “We use the 3D measurement of the wave magnetic field to derive the total power spectral density”. Does that mean that the authors estimate the power of the total magnetic field intensity?

6) Figure 1: Generally, the solid lines are very hard to recognize. It would be better for the reader if the authors would plot the lines bold (thicker) and added a legend describing what they represent (e.g., 0.5*fce).

7) Line 131: A part of the plasmaspheric hiss shown in figure 1c corresponds to L values higher than 4.6 (left side of 1c). This is of course not surprising since the O'Brien model is empirical and thus expected to exhibit deviations from reality. Nevertheless, this raises a question of what portion of the following statistics is due to plasmaspheric hiss instead of chorus and exohiss and how much uncertainty the results have.

8) Lines 142-143: Is this a linear or logarithmic averaging? Since the values span several orders of magnitude one could assume that a linear averaging favors the higher values.

9) Lines 145-146: Is kappa the same for RBSP and Cluster? If this is linked to the resolution of the data, I would assume it is not the same; nevertheless, only one correction factor is mentioned here. Please clarify.

10) Line 161: There is only figure 1 and not figure 1a in the supplementary material.

11) Lines 336-337: If the 11 RE is a hard threshold, how the authors can be sure that the data used are always inside the magnetopause?

12) Lines 348-371: This is confusing. Are the measurements indeed cross-calibrated to obtain the results shown in figure 8 or do the authors show the comparison of the native measurements suggesting that the divergence of the two mission measurements are really close anyway? If the latter is true, then the term "inter-calibration" should not be used here.

Reviewer #4

(Remarks to the Author)
I co-reviewed this manuscript with one of the reviewers who provided the listed reports. This is part of the Nature Communications initiative to facilitate training in peer review and to provide appropriate recognition for Early Career Researchers who co-review manuscripts.

Version 1:

Reviewer comments:

Reviewer #1

(Remarks to the Author)
Second Review of “Prevailing Dayside Occurrence of Lower Band Whistler Mode Chorus and Exohiss” by Santolik, Kolmasova, Taubenschuss and Hanzelka

I find the paper acceptable for publication if the authors give credit to others who have reported similar chorus features. I have no further comments.

Reviewer #2

(Remarks to the Author)
After carefully reading the revised manuscript and the Response to my previous comments, I regret to write that my position concerning this manuscript is unchanged. The main results of the present manuscript (emphasized in the Title and Abstract) concern the global features of chorus wave occurrences as a function of MLT (local time), wave amplitude, and latitude, and they were already discovered 15 years ago by Li et al. (2009). The "prevailing dayside occurrence" of moderate amplitude chorus waves (emphasized in both the Title and Abstract of the present manuscript) was first discovered by Li et al. (2009)

and confirmed by Agapitov et al. (2013). The shift of chorus wave occurrences toward the postmidnight sector for wave amplitudes above 100 pT (also emphasized in the Abstract of the present manuscript) was also first discovered by Li et al. (2009). These important discoveries justified the publication of the results of Li et al. (2009) in Geophysical Research Letters (a journal roughly equivalent to PRL in the limited field of geophysics).

In the revised manuscript, the differences between the details within these global features of chorus wave occurrences, like in this or that individual MLT bin, and the different numbers of years of data used, and the details of the different data analysis procedures used in each study, are extensively discussed. But at the same time, the strong similarities of the main, global features of chorus wave occurrences shown in the present Figure 4 with the previous results in Figure 3 of Li et al. (2009) and Figure 3(b,f) of Agapitov et al. (2013) are not acknowledged. This can easily give the misleading impression that the main results of the present manuscript (on the global features of chorus wave occurrences) are new, which is not exact.

The present study, performed 15 years later, provides a more precise knowledge of the very fine details of the chorus wave occurrence distribution in each individual MLT (local time) bin than in Li et al. (2009) or Agapitov et al. (2013), but this incremental improvement is clearly insufficient to justify a publication in Nature Communications. It does not provide a significant improvement to our knowledge of the physical processes leading to chorus wave generation.

Finally, for simulations of the effects of chorus waves on the dynamics of radiation belt electrons (the "critical role" of chorus waves mentioned in lines 11-12 of the Abstract), the only important parameter is the CHORUS WAVE POWER DISTRIBUTION (e.g., see Thorne et al. 2013, Wong et al. 2024 doi:10.1029/2023JA031607) and NOT the CHORUS WAVE OCCURRENCE DISTRIBUTION discussed in the present manuscript. Various models of this chorus wave power distribution have been provided by Meredith et al. (2012, 2020) and Agapitov et al. (2018) based on the same data from the Van Allen Probes and Cluster missions. These three recent studies did not examine anymore the "wave occurrence distribution" in isolation, contrary to what is done in the present manuscript, simply because this "wave occurrence distribution", alone, is known to be unimportant for simulations of the dynamics of radiation belt electrons.
Therefore, the present manuscript deserves to be published in a more specialized journal, like Journal of Geophysical Research (Space Physics).

(1) The three main results of the present manuscript (presented in Figure 4), concerning the global features of chorus wave occurrences as a function of MLT (local time), wave amplitude, and latitude, presented in Figure 4, were already discovered 15 years go by Li et al. (2009).

- First, it is clear in Figures 3(a) of Li et al (2009) for moderate amplitude waves (Bw=10-30 pT) at Latitudes < 10 deg that the peak occurrence rate patch (yellow-red) is located at 7-13 MLT at L > 7 and at 8-17 MLT at L = 5-7, i.e., on the dayside.
It is also clear in Figures 3(b) of Li et al (2009) for moderate amplitude waves (Bw=10-30 pT) at Latitudes 10-25 deg that the peak occurrence rate patch (yellow-red) is also located on the dayside, at 8-13 MLT at L > 7 and at 7-17 MLT at L = 5-7.

- Second, it is also quite clear from Figure 3(a) of Li et al. (2009) that when wave amplitude Bw increases from Bw=10-30 pT to Bw = 30-100 pT to Bw > 100 pT, the peak occurrence rate patch (yellow-red) at L = 5-7 moves from the dayside (8-17 MLT) to the postmidnight (midnight-to-dawn) sector (0-8 MLT) at latitudes < 10 deg.

- Third, comparing Figures 3(a) and 3(b) from Li et al (2009), it is clear that the shift of occurrence rates from dayside to the postmidnight (midnight-to-dawn) sector present at latitudes < 10 degrees, is not present at higher latitudes > 10 degrees.

These three major discoveries from Li et al. (2009) on the global features of chorus wave occurrences as a function of MLT, wave amplitude, and latitude, are the SAME as the THREE MAIN RESULTS of the present manuscript (shown in Figure 4 and noted in the Title itself and the Abstract).

Figures 3(b,f) from Agapitov et al. (2013) similarly show a broad peak of chorus wave occurrences on the dayside (at 9-19 MLT) at L = 5-8 when considering all such waves measured by Cluster, i.e., mainly waves of moderate amplitudes. As shown in Figures 3(a,e,i) of Agapitov et al. (2013), the corresponding average amplitudes of such dayside chorus waves in their Figures 3(b,f) are Bw = 10-20 pT, far above the instrument noise level (contrary to the statements in lines 385-386 of the revised manuscript) and similar to the "moderate amplitude waves" of the present manuscript and of Li et al. (2009).

(2) The manuscript (especially lines 379-392), as well as the response, are confusing. They focus on some fine details in the figures of Li et al. (2009), on some presumed limitations of the datasets of Li et al. (2009) or Agapitov et al. (2013), and on the obtained precise percentage level of chorus wave occurrences (this level, however, is known to vary in time and over different solar cycles, so that one cannot expect to get the same level with different datasets covering different time spans). There are also instances where previous works are strongly minimized without supporting evidence (lines 379 and 385-386).

However, the main facts remain: THE MAIN, GLOBAL FEATURES ARE THE SAME AS IN LI ET AL. (2009) AND AGAPITOV ET AL. (2013). At low latitudes, both the "prevailing dayside occurrence" (8-17 MLT) of moderate-amplitude chorus waves and the shift of this broad peak of chorus wave occurrences to the postmidnight (midnight-to-dawn) sector (0-8 MLT) at high wave amplitude were already discovered by Li et al. (2009). At high latitudes, the absence of this MLT shift of the peak occurrences from 8-17 MLT to 0-8 MLT as the wave amplitude increases, was also discovered by Li et al (2009). The dayside (9-19 MLT) peak of occurrence of moderate-amplitude chorus waves at L = 5-8 was also confirmed by Agapitov et al. (2013) at both low and high latitudes.
Of course, the more limited statistics of Li et al. (2009) or Agapîtov et al. (2013) did not allow them to provide a very precise

MLT distribution of occurrences in each individual MLT bin, as done in the present manuscript. But these improvements provided in the present manuscript are merely incremental improvements, compared with the important discoveries of Li et al. (2009), and such incremental improvements are clearly insufficient to justify a publication in a high-level journal like Nature Communications.

(3) Lines 54-56, it is written that "Another simplification is that intervals with instrumental noise are simply included in the long term averages, as none of the existing models properly introduces detection thresholds nor the occurrence rates of chorus and exohiss".
This unjustified statement should be suppressed or seriously reformulated, because it is false. Most existing chorus wave models (e.g., from Li et al., (2009), Meredith et al. (2012, 2020), Agapitov et al. (2013, 2018)) properly incorporated occurrence rates and used a detection threshold in wave amplitude at 1-10 pT and/or additional thresholds leading to a high signal-to-noise ratio.
For instance, Meredith et al. (2012) noted the following about their chorus wave model (in their Section 6):
"Differences in sensitivity between the instruments can affect the average intensities but are limited to very low intensity levels when the spectral intensity is below the threshold for detection in one or more of the instruments. The effect is negligible when the wave power is well above the threshold for detection for all of the instruments, which roughly equates to wave power above 1 pT2 [squared]. Since chorus wave power below 1 pT2 is insignificant and has an insignificant affect on diffusion rates our model captures all of the important chorus wave power."

(4) Lines 262-263: "A very important aspect of our study is that we increased the statistical significance of our results by combining the data of the Cluster and Van Allen Probes missions."
This "very important aspect of the present study" is not new at all: Agapitov et al. (2018) already combined the data of the Van Allen Probes and Cluster missions to provide a model of time-averaged chorus wave power, naturally incorporating chorus wave occurrences.

(5) Using an empirical plasmapause model (as in the present study) is not very accurate for determining the plasmapause and the L-shell domain where chorus waves are presumed to be present at each time. It would be much better to use actual plasmapause crossings available from Van Allen Probes density measurements.

(6) Lines 264-269. The authors only consider chorus waves inside a domain comprised between the model plasmapause plus 1 Earth's radius (RE), and the field line at an equatorial distance of 6 RE. This suggests that they exclude many quiet times (i.e., moderate amplitude) chorus waves located at 5.5-7.0 RE, potentially leading to an important bias in the provided MLT distribution of moderate amplitude chorus waves in the outer radiation belt.

Reviewer #3

(Remarks to the Author)
Dear authors,

thank you for the thoroughly revised manuscript and your detailed responses to the reviewers' comments.

I now consider the manuscript acceptable for publication.

Ioannis A. Daglis

Reviewer #4

(Remarks to the Author)
I co-reviewed this manuscript with one of the reviewers who provided the listed reports. This is part of the Nature Communications initiative to facilitate training in peer review and to provide appropriate recognition for Early Career Researchers who co-review manuscripts.

Version 2:

Reviewer comments:

Reviewer #2

(Remarks to the Author)
Overall, this very significantly revised version of the original paper provides much more useful and interesting results on the chorus wave power distribution and on time delays between chorus wave power occurrences at high wave power. These distribution should be useful for better modeling the effects on electron fluxes and understanding the generation of chorus waves.
However, several important points should be improved before a possible publication. The most important problem is with new Figure 3, where the plotted time delays may not always be representative of actual delays (suggestions for improvements are provided below). The suggested impact on long-term simulations of radiation belts should also be discussed more accurately.

1. New Figure 1, showing PDFs of chorus amplitude, is an important addition for undestanding chorus wave generation and

propagation mechanisms. You could further note in the text (near Line 108) that the the long-term average (white line) provided in Panel (e) should be used in long-term radiation belt quasi-linear simulation and that, although this provided long-term average roughly agrees with previous statistics (Agapitov et al. 2018), it is more accurate and available up to higher latitudes thanks to the larger dataset used. This distribution in Panel (e) may also account for the apparent presence of significant chorus wave ducting in the noon sector recently inferred from ELFIN measurements of the precipitating-to-trapped electron flux ratio (Zhou et al. 2025 https://doi.org/10.1029/2025JA034117).

2. I have some concerns about new Figure 3 and suggestions of improvements.

As concerns Figure 3, the Authors should note in the Main text that based on the typical orbital speed, spacecraft should traverse the chorus source region in 100-200 s for typical "chorus source region" transverse sizes (at 0.5 correlation) of 400-800 km determined using Van Allen Probes data (Agapitov et al. 2021 https://doi.org/10.1029/2020JA028998, Z. Zhang et al. 2021 https://doi.org/10.1029/2021JA029569). This implies that the present observations are usually not independent for time delays less than 200 s, which probably explain the power-law shape in Figure 3 for delays less than 200 s. Agapitov et al. 2021 (https://doi.org/10.1029/2020JA028998) further noted that the typical wider "chorus active region" size (defined as the maximal distance at which chorus waves are observed simultaneously in at least 50% of the observations with squared amplitudes higher than 9 pT^2, see their Fig.7d) is 4000-8000 km at 8-14 MLT in the transverse direction, corresponding to time delays of 15-35 min. This implies the presence of additional correlations between spacecraft observations of chorus waves, up to delays of 2000 s. This order of value is in good agreement with the range 10-2000 s of time delays in Figure 3 where the power-law slope remains constant. Above 2000 s delays, the negative slope often decreases before increasing again above 2 10^4 s in Figure 3. The Authors already wrote about the spatial chorus correlation (Lines 522-524 in Methods Section), but this important point should also be mentioned in the main text to explain Figure 3 based on typical chorus source region and chorus active region sizes determined using the same Van Allen Probes dataset as in the present work.

However, for time delays longer than about 10^4 s, a Van Allen Probe has already travelled far away from its initial (L,MLT) region of measurements and the recurrence periods between consecutive measurements at a same L or at a same (L,MLT) should start to play an important role in the distribution of time delays. Such recurrences should introduce additional correlations between measurements and might account for the more rough power-law shape above 10^4 s. Cluster spacecraft have longer orbital periods but more inclined orbits, so that recurrences at a same L or (L,latitude) should also start to play a role above 10^4 s. The presence of such recurrences at long time delays suggests that the results in Figure 3 should be taken with caution above 10^4 s. They likely do not fairly represent the distribution of actual time delays at a given, fixed L or (L,MLT) or (L, latitude).
Therefore, I would suggest to stop the curves in Figure 3 as dashed lines at 10^4 s.
It would also be important and useful to plot additional panels in Figure 3, showing the same distribution of delays in fixed (L, MLT) ranges (say +/- 0.5 R_E in L and +/- 1 MLT) at 11-13 MLT and 0-2 MLT, above 10^3 to 10^5 pT^2 (2 x 3 = 6 additional panels).
This could provide more useful distribution of delays for radiation belt simulations.
You may consider keeping only these new panels instead of the present panels, because the present panels are less easy to interpret and probably provide less reliable distributions of delays in a given L or (L,MLT) region (the latter are the most interesting for simulations and for understanding chorus generation mechanisms).

3. The statements in Lines 380-382 and 390-395 and are excessive and should be seriously amended. The present dataset consists of 0.5-s-averaged wave power (VAP) or 4-s-averaged power (Cluster). Actual chorus wave elements mainly consist of trains of relatively short intense wave packets lasting less than 10-20 wave periods (0.005-0.1 s), with a roughly power-law shape PDF of wave packet power at 0.1-1 nT (X. Zhang et al. 2021 https://doi.org/10.1029/2021JA029330). Therefore, intense wave packets of 10^4 pT^2 or 10^5 pT^2 should be much more frequent than 0.5-s or 4-s bursts of the same power at a given L, and occur after much shorter time delays than in Figure 3. In other words, wave packet variability, including wave phase and amplitude variability and radial variability, may still allow quasi-linear theory to remain applicable (Artemyev et al 2025 https://doi.org/10.1007/s11214-025-01144-y, Hanzelka et al. 2025 https://doi.org/10.1029/2025GL116883).

Reviewer #3

(Remarks to the Author)
Although the study is primarily a statistical characterization of wave amplitude distributions and spatiotemporal patterns and does not directly study chorus generation mechanisms at the microphysical level, it nevertheless advance knowledge relevant to chorus generation physics through several of its results.

1. A major new finding is the clear separation of two regimes as a function of wave amplitude threshold:
- Low-amplitude waves occur on the dayside, noon-centred, across all latitudes up to ±50°.
- While for high-amplitude waves the occurrence shifts sharply to the post-midnight equatorial region (low MLat).
This amplitude-dependent shift, documented quantitatively for the first time with this statistical weight (~26 years of data), directly constrains the two distinct generation populations described by the authors.
Prior work (THEMIS, Li et al. 2009; Cluster, Agapitov et al. 2013) had lower coverage and noted a dayside peak, but could not resolve this amplitude-dependent transition from dayside to post-midnight dominance with the same precision.

2. The discovery that time delays between detections follow a power-law (not Poisson) distribution is a genuinely new physical constraint. A Poisson distribution would indicate independent, memoryless events — consistent with steady or quasi-random linear growth. A power law reflects clustering across multiple timescales, implying that wave generation

events are not independent but mutually correlated — consistent with bursty, injection-driven cyclotron instability where one substorm injection triggers a wave burst, whose envelope modulates subsequent bursts. Previous studies noted the bursty character qualitatively; this is the first large-scale statistical quantification of the inter-event time distribution and its dependence on amplitude.

3. The substorm–chorus link has been confirmed and extended.

4. The rising occurrence of low-amplitude waves on the dayside near solar maxima, points to a solar wind–controlled generation pathway for the dayside wave population that is distinct from the substorm-driven post-midnight pathway.

However, there are also some methodological and conceptual shortcomings. In particular:
1. The most significant scientific limitation - acknowledged by the authors - is that chorus and exohiss are analysed together as a single population throughout the study. It is a fundamental problem, because the two wave types have very different generation mechanisms. Binning them together blurs physically distinct phenomena.
2. The dataset is heavily non-uniform in magnetic latitude coverage. The authors note this but claim the statistics remain adequate. However, for heavily constrained subsets (e.g., intense waves during specific solar cycle phases at high latitudes), the coverage degrades severely, causing large random fluctuations that the authors acknowledge as a real limitation.
3. The L parameter and the plasmapause boundary are calculated using a pure dipole magnetic field, which is a recognised oversimplification.
4. The Van Allen Probes operated during the declining phase of Solar Cycle 24 only (~2012–2019), whereas the Cluster mission spans Solar Cycles 23 and 24. The two datasets therefore sample systematically different solar activity distributions.

Nevertheless, the manuscript merits publication despite its shortcomings.

Version 3:

Reviewer comments:

Reviewer #2

(Remarks to the Author)
The Authors have well addressed all comments.
The presented results in Figures 3-4 are both new and important. These figures provide for the first time the distribution of time delays between observations of intense chorus waves and its variations with MLT and demonstrate solid power-law behavior. These results will be useful for advancing our understanding of chorus wave generation, by providing new constraints for generation models, and may also be useful for improving the accuracy of radiation belt modeling over moderately long time periods. Other results, in Figures 1-2 and Supplementary Information, usefully complement previous works by providing more information on chorus power and occurrence distributions at high latitudes of 30 to 60 degrees, where measurements from all missions are sparse.
Therefore, the paper now deserves publication in Nature Communications.

Open Access This Peer Review File is licensed under a Creative Commons Attribution 4.0 International License, which permits use, sharing, adaptation, distribution and reproduction in any medium or format, as long as you give appropriate credit to the original author(s) and the source, provide a link to the Creative Commons license, and indicate if changes were made.
In cases where reviewers are anonymous, credit should be given to 'Anonymous Referee' and the source.
The images or other third party material in this Peer Review File are included in the article's Creative Commons license, unless indicated otherwise in a credit line to the material. If material is not included in the article's Creative Commons license and your intended use is not permitted by statutory regulation or exceeds the permitted use, you will need to obtain permission directly from the copyright holder.
To view a copy of this license, visit https://creativecommons.org/licenses/by/4.0/

Response to Reviewer #1

We thank the reviewer for her/his time spent on the review, and for her/his constructive comments. The review report is quoted in *blue italics*, followed by our point-by-point responses. The line numbers refer to the revised manuscript with tracked changes.

Review of "Prevailing dayside occurrence of lower band whistler mode chorus and exohiss" by Santolik, Kolmasova, Taubenschuss and Hanzelka

The paper is very well written, contains important new information and should be published in Nature Communicates after some minor corrections have been made.

We have prepared a major revision of the manuscript based on the comments of all the reviewers.

Comments

Abstract lines 12 and 13: The use of 4 Cluster spacecraft data and two Van Allen Probes data is a case of oversampling since the 4 Cluster spacecraft collect essentially same chorus and exohiss data.

The separation distances of the four Cluster spacecraft varied during the analyzed time interval, as it is shown on this figure taken from Escoubet et al. 2022, doi: 10.1029/2021JA029474

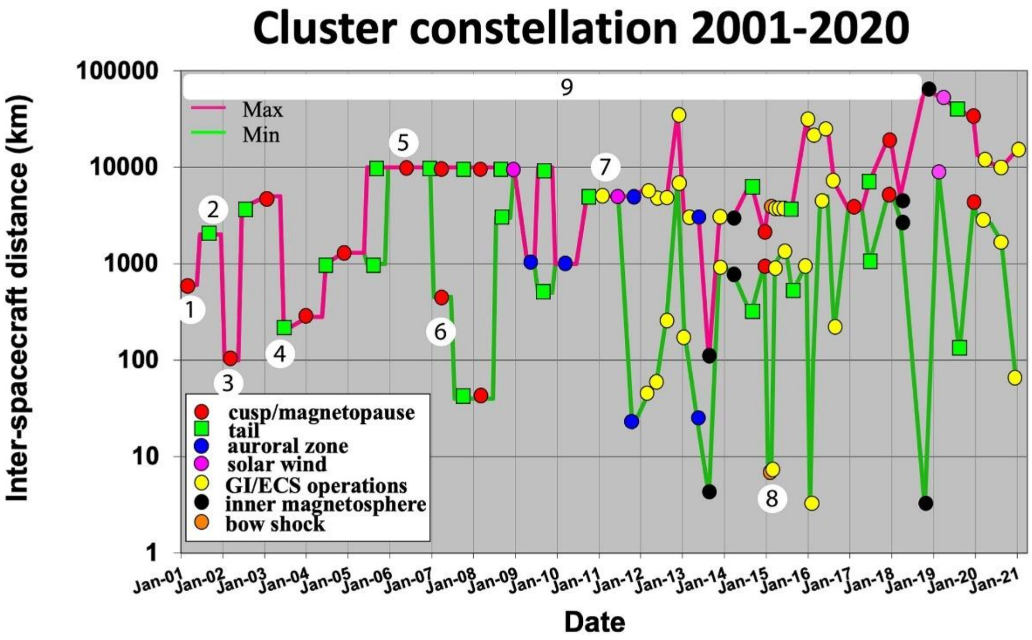

Previous studies (doi:10.1029/2002GL016178, 10.5194/angeo-22-2555-2004) showed that, in order to observe the same parallel propagating chorus elements, the separation distance perpendicular to the field line must be typically on the order of 100 km or less. In this sense, oversampling mentioned by the reviewer occurs very rarely, with negligible influence on the conclusions of this manuscript. Analysis of data from separate spacecraft yields consistent results but with lower sampling statistics. We have newly added a discussion of this problem on lines 450-469.

Line 17. "The peak of chorus occurrence being located symmetrically about local noon" and Figures 3a and 3b. This distribution is most likely dominated by the lowest intensity waves of your study. Please use high time resolution chorus/hiss data taken at 1700 local time to examine 10 to 20 samples to confirm that these low intensity events are indeed chorus and hiss and report this in the paper. Right now it is uncertain what this symmetry is due to.

We have examined the high-resolution measurements on the dusk side of the main occurrence peak in (now renumbered) Figures 4a and 4b and found both chorus and exohiss. Examples are given in Supplementary Figures 17 and 18. The distribution of amplitudes is highly dispersed and heavy-tailed, as we state on lines 51-57. Therefore, higher amplitudes occur also in this region. Vice-versa, the distribution is dominated by low amplitudes everywhere in the parametric space but a larger fraction of high amplitudes occurs in the equatorial postmidnight sector.

Based on this reviewer's comment, we have realized the importance of showing representative examples of high resolution data also in the main paper, linked to the cases from Figure 1. Consequently, we have added a new Figure 2 containing these examples.

Figure 3 is not in contradiction to the prevailing scenario of chorus generation. There are three predominant sources of chorus: one is local midnight injection of anisotropic ~10 to 100 keV substorm electrons (your reference 1). A second source is when the drifting energetic electron cloud crosses the dawn boundary, the enhanced thermal plasma density leads to larger growth rates of chorus and thus the original name "dawn chorus".

We have rewritten the first two paragraphs of the Discussion section on lines 373-392 to include these suggestions.

The third is solar wind pressure pulses on the dayside magnetosphere (see JGR, 117, A00L12, 2012 doi?10.1029.20011JA017180; JGRSP, 121, 2016 doi:10.1002/2016JA022499). It should be noted that in the year long survey (much shorter than yours), in JGR, 82, 32, 5112-5128, 1977 the midnight to dawn sector chorus was much weaker than the dayside chorus. So these scenarios generally fit with your Figures 1c and 1d.

This is now discussed in detail (and recommended references included) in the third paragraph of the Discussion section on lines 393-405.

Lines 17-19. I think this is in agreement with the 1977 paper mentioned above. I will say more about Figure 3e and 3f later.

This is now discussed in the third paragraph of the Discussion section on lines 393-405.

Lines 29-40. Very nice set of statements. This needs to be told to the readership. Thank you.

We thank the reviewer for this encouraging comment.

Line 37. I suggest adding a reference here. JGRSP, 125, 2020 doi.org/10.1029/2020JA028090.

Added as ref 22 on line 39.

Lines 61-65. This may be too strong of a statement. I think it is only for the lowest amplitude chorus/exohiss waves.

This statement is now softened by adding that our results include all detectable events, line 63

Figure 3. Please indicate the differences in the 6 panels in the Figure. This will be more convenient for the readership instead of trying to dig it out of the caption.

We have added a detailed description of the differences in the 6 panels of the now renumbered Figure 4. The additional amplitude constraints are now given in Figure 4 and in the text for all the panels (lines 316-331).

Lines 130-133. Please say something about Figures 3e and 3f. This very high intensity chorus/exohiss at midnight seems peculiar. I assume that there are very few events here? Perhaps a magnetic storm where the particle injection was extreme, giving extreme electron anisotropies? This high amplitude chorus is at odds with the intensities found in your reference 1. Please take a look using high time resolution data at this event.

We have verified that this postmidnight low latitude bin (0-2h MLT, 0-5deg |MLat|) really contains many high amplitude events. Examples are given in Supplementary Figure 17a-b. Without additional constraints this bin contains 352,994 measurements, with 54% occurrence rate, originating from 287 different days. With an additional constraint of waves with effective squared amplitudes above 10^4 pT, this bin still shows 22,272 measurements from 170 different days and an occurrence rate of 3.4%, and the additional constraint of squared amplitudes above 10^5 pT results here in 2,624 measurements from 92 different days and an occurrence rate of 0.4%. We now note it on lines 478-491 in the Discussion section. It might have been missed in reference 1 because of this low occurrence rate.

Line 275. Please add the reference JGR 114, A03207, 2009 doi:10.1029/2008JA013353 here.

Added as ref 50 on line 397.

Line 280-281. This is a bit of an overstatement. Only for low intensity chorus and exohiss?

We have softened this statement, it is now moved to the first sentence of the Discussion section on lines 373-375. However, not only low intensity chorus or hiss can be found in the main noon-centered occurrence peak. Examples are given in Supplementary Figures 17 and 18. The distribution of amplitudes is highly dispersed and heavy-tailed, as we state on lines 51-57. Therefore, higher amplitudes occur also in this region.

Lines 295-297. I agree. But please look at some of your low intensity events to make sure what they are. Also report it in the paper.

All survey mode intensities in the new Figure 2 in the main paper are rather low, within a range of 12-20 pT for all panels a-f of Figure 2, and they are both chorus and exohiss, as we now discuss in lines 470-477. Other examples of low intensity chorus and hiss waves are given in Supplementary figure 18.

Response to Reviewer #2

We thank the reviewer for her/his time spent on the review. The review report is quoted in *blue italics*, followed by our point-by-point responses. The line numbers refer to the revised manuscript with tracked changes.

This manuscript presents a study of the occurrence rate of lower band whistler mode chorus and exohiss waves in the inner magnetosphere of the Earth, based on a combination of well inter-calibrated data from the Cluster four-spacecraft mission and from the Van Allen Probe two-spacecraft mission.

The main results of the manuscript are that:

(a) the overall occurrence rate of chorus waves (of both low and high amplitudes, but mainly low amplitudes) has a large peak at 8-16 MLT, where occurrences are much larger than at 22-6 MLT,

(b) the peak occurrence rate of chorus waves moves from 8-16 MLT to 0-6 MLT at low latitudes as chorus wave amplitude increases from < 30 pT to > 100 pT, but remains at 8-16 MLT at high latitudes.

The problem with this manuscript is that these two main results, (a) and (b), are not new at all, as easily demonstrated below. Exactly the same results have already been published based on THEMIS and Cluster statistics. Therefore, the present manuscript does not contain sufficient new results to be published in Nature Communications.

We respectfully disagree with the reviewer about this list of main results of our manuscript, which limits the results to the descriptions of the positions of occurrence peaks of chorus and omits our quantitative description of the occurrence rates of lower-band chorus and exohiss waves. Our abstract in fact summarizes the main results differently: "…we determine occurrence probability of lower band chorus and exohiss, obtaining a peak value above 80% around the local noon, within a large range of latitudes. This can only be reconciled with the traditional picture of postmidnight chorus considering a selection effect for amplitudes above 100 pT, showing however, only small occurrence probability below 4%." Our ability to properly quantify the occurrence rates in a large range of magnetic latitudes and under different constraints for wave amplitudes is a new, important, and potentially impactful result of our study.

We disagree with the statement of this reviewer that our results are not new. Our results are new, based on a combination of data of multiple spacecraft with different orbits. The previously published papers that the reviewer points out are undoubtedly admirable pieces of work but, as we show below, they do not even contain the items (a) and (b) that the reviewer selected from our results, nor do they contain our quantitative results.

1. It is found in the present manuscript that (a) the occurrence rate of chorus waves of low amplitudes (<30 pT) has a large peak at 8-16 MLT, where occurrences are much larger than at 22-6 MLT.

Unfortunately, exactly the same result has already been found by Li et al. 2009 (doi:10.1029/2009GL037595) using chorus wave Filter Bank Data statistics from THEMIS spacecraft (it consists of 4-second wave data as in the submitted manuscript).

Figure 3(left column) from Li et al. 2009 shows the same peak of occurrences of chorus waves at 8-14 MLT, at both low (<10 degrees, top panel in left column) and high (10-25 degrees, bottom panel in left column) latitudes, for waves of low amplitudes < 30 pT, exactly as in Figures 3(a-d) from the present manuscript.

This comment contains a sequence of statements, which should be properly put in the context of the actual contents of the reviewed manuscript.

a) First of all, it must be noted that the nice and insightful paper of Li et al. 2009 already was on the list of references of the submitted version of the present manuscript and that we are aware of the results and conclusions of this paper. We detail the differences below.

b) Looking at the source dataset, Li et al. 2009 used magnetic field amplitudes from the Filter Bank Data product from 5 THEMIS spacecraft collected at magnetic latitudes within 25° from the equator during approximately 1.5 years. The limited frequency resolution of this data set (see their Fig. 1) left the authors with a few very wide frequency channels to cover the frequency range between 0.1 f_{ce} and 0.8 f_{ce}. As the text of the Li et al. 2009 paper suggests ("...if 0.1 f_{ce} is larger than 320 Hz, chorus is assumed to be confined to the top two filter bank bands..."), this procedure may hypothetically lead to mixing with other intense wave modes, like linearly polarized equatorial noise. There is no possibility of selecting right-hand polarized whistler-mode waves from the THEMIS Filter Bank Data set, because only average wave amplitudes were measured, and the wave polarization was not analyzed. The dataset we analyzed is therefore not as similar as suggested by the reviewer as it covers more than 10 times longer time interval of measurements, a larger range of latitudes up to 60°, with a finer frequency resolution, and unlike in THEMIS Filter Bank Data, the measured polarization always allows us to select the desired wave mode.

c) As concerns the results, the reviewer refers to Figure 3 (left column) from Li et al. 2009, noting that "…it shows the same peak of occurrences of chorus waves at 8-14 MLT … for waves of low amplitudes < 30 pT, exactly as in Figures 3(a-d) from the present manuscript." The peak in Figure 3 (left column) from Li et al. 2009 is clearly centered well before noon, around 8-10 MLT, while our results show an approximately symmetrical peak around 12 MLT. The reviewer misses the point that we observe the peak centered at 12 UT consistently in a large range of magnetic latitudes up to 55°. Our 5 ° wide latitude bins allow us to see decreased occurrence rates down to 10-15% around the geomagnetic equator for amplitude thresholds above 10 pT. We also observe much larger occurrence rates above 80% for lower amplitudes. None of these results was obtained by Li et al. 2009.

d) The source of the observed differences can be probably tracked down to the limited data set used by Li et al. 2009. The question now is how important the observed differences are. We are convinced that our results are substantially different. The symmetric peak around local noon extending within a large range of latitudes opens new possibilities for testing the theories of origin of the dayside chorus, different from those, which would be needed to explain the pre-noon occurrence peak found by Li et al. 2009. Several times larger occurrence rates found in our study (approaching 100%) , as well as the newly characterized latitudinal distribution will lead to different estimates of the effects of these waves through wave particle interactions than those, which would result from the analysis of Li et al. 2009.

Unsurprisingly, a similar peak of occurrences near 12 MLT when keeping ALL chorus waves (i.e., mainly low amplitude waves), very similar to Figure 3(a) of the present manuscript, has also been shown in

Figure 3(b,f) from Agapitov et al. 2013 (doi:10.1002/jgra.50312), at both low latitudes (<15 degrees) and high latitudes (>15 degrees), based on the same data from Cluster spacecraft used in the present manuscript.

Agapitov et al. 2013 is a paper with many different results, containing also a correction of previously published erroneous results by Agapitov et al. 2011. However, in the disagreement with the reviewer's comment, we are convinced that this paper does not contain the results that we describe in the present manuscript, as we detail below:

e) The data set is substantially different: Agapitov et al. 2013 use 10 years of measurements of one of the Cluster spacecraft (Cluster 4). Our data set in the present study covers more than 19 years of measurements of all four Cluster spacecraft between 7 January 2001 and 30 April 2020 and the entire operational period of both Van Allen Probes. Concerning the Cluster data only, the data set in the present paper is therefore approximately 8 times larger than that of Agapitov et al. 2013. The poor coverage of the equatorial region in the paper of Agapitov et al. 2013, which negatively influenced the quality of their determination of occurrence rates at low latitudes is cured in our study by using the large low-latitude dataset from Van Allen Probes.

f) The results are substantially different and the reviewer's comment "Unsurprisingly, a similar peak of occurrences near 12 MLT when keeping ALL chorus waves (i.e., mainly low amplitude waves), very similar to Figure 3(a) of the present manuscript, has also been shown in Figure 3(b,f) from Agapitov et al. 2013 …, at both low latitudes (<15 degrees) and high latitudes (>15 degrees)," is probably due to some misunderstandings.

- First, Agapitov et al. 2013 do not seem to use any thresholds based on characteristics of instrumental noise in their Fig. 3, which is an essential step in determination of occurrence rates. Noise thresholds are strongly frequency dependent, and depend also on time, as the instruments degrade during the mission. Fig. 3 of Agapitov et al. 2013 therefore may erroneously contain contribution from detections of instrumental noise at separate frequencies in the analyzed band. According to their description, they only use a fixed threshold of 1 pT for the frequency integrated amplitude to determine the occurrence rate. The reviewer's statement "…when keeping ALL chorus waves (i.e., mainly low amplitude waves), …. has also been shown in Figure 3(b,f) from Agapitov et al. 2013" therefore is not consistent with the text of Agapitov et al. 2013.
- Second, looking at Figure 3(b) of Agapitov et al. 2013 huge fluctuations of at least one order of magnitude can be noticed between occurrence rates in neighboring bins. This is surely not real, these fluctuations, although not explained in their text, are most probably an artifact resulting from poor statistics of their low latitude measurements. Moreover, they found the largest occurrence rate values in the afternoon sector, therefore no "peak of occurrences near 12 MLT" can be inferred from this figure. Figure 3(f) of Agapitov et al. 2013 is more relevant as it contains more data to actually show a pronounced peak of occurrence centered around 15-16 MLT, while the occurrence rates decrease several times near 12 MLT. So again, no "peak of occurrences near 12 MLT". It is therefore evident that just a simple observation of "Figure 3(b,f) from Agapitov et al. 2013" indicates a potential misunderstanding.
- Third, if we attempt to compare "Figure 3(b,f) from Agapitov et al. 2013" with our results, it is clear that "Figure 3(a) of the present manuscript" (Fig. 4a in the revised version) is not the right

choice, because the amplitude thresholds are different, as noted above. The closest would probably be our Fig. 4b, which uses an additional threshold of 1 pT, on the top of the noise threshold, described in our text, while Agapitov et al. 2013 only uses the 1 pT threshold without removing the noise detections. The comparison clearly shows a significant difference in the positions of the occurrence peak, our result being centered around 12 MLT, compared to 15-16 MLT, with the occurrence rates decrease several times near 12 MLT in Agapitov et al. 2013. A possible source of the observed differences is, besides a flawed occurrence analysis procedure of Agapitov et al. 2013, their plasmapause model, which may cause a contamination of their occurrence rates at lower L values by plasmaspheric hiss or by lightning whistlers in the afternoon region.

2. It is found in the present manuscript that (b) the peak occurrence rate of chorus waves moves from 8-16 MLT to 0-6 MLT at low latitudes as chorus wave amplitude increases from < 30 pT to > 100 pT, but remains at 8-16 MLT at high latitudes.

Unfortunately, exactly the same results have already been found by Li et al. 2009 using THEMIS data.

As shown in Figure 3(top row, from left to right) from Li et al. 2009, at low latitudes (< 10 degrees) the peak occurrence rate of chorus waves is shifted from 8-16 MLT for wave amplitudes < 30 pT to 0-6 MLT for wave amplitudes > 100 pT, exactly as in Figure 3(c-f) from the present manuscript. At the same time, Figure 3(bottom row, from left to right) from Li et al. 2009 shows that the peak occurrence rate at high latitudes (10-25 degrees) remains at 8-14 MLT as the wave amplitude increases from 10-30 pT to > 100 pT, exactly as in Figure 3(c-f) of the present manuscript.

(g) The reviewer repeats that "…exactly the same results have already been found by Li et al. 2009 using THEMIS data…". The initial part of our response therefore still applies here: we disagree with the reviewer about this list of main results of our manuscript, and we disagree with the statement that our results are not new. Also, points a) and b) of our response to the first comment of reviewer #2 still apply here. Li et al. 2009 did a very good job in extracting relevant pieces of information from the limited data set of the Filter Bank Data product from 5 THEMIS spacecraft collected during 20 months. However, they couldn't change the limited frequency resolution of the instrument, nor could they change the near-equatorial spacecraft orbit at magnetic latitudes within 25° from the equator. Moreover, their dataset also implied a limited coverage of infrequent events with larger amplitudes.

(h) Responding now only to the reviewer's choice of results mentioned in this comment, we note that it starts by the same statement, to which we already responded in point c) of our response to this reviewer. We can only repeat that the peak in Figure 3a (left column) from Li et al. 2009 is clearly centered before noon, around 8-10 MLT, while our results in Figure 4c of the present manuscript are significantly different, showing an approximately symmetrical peak around noon, consistent over a large range of latitudes. We may also notice that Figure 3a (middle column) of Li et al. 2009 shows similar distributions as those in the leftmost column of the same figure. The maximum occurrence is again between 7 and 11 MLT, and the main differences are larger fluctuations between neighboring bins, caused undoubtedly by a lower amount of data in the statistics for larger amplitudes in the middle column. These results change only slightly for latitudes between 10° and 25° in Figure 3b (left and middle column) of Li et al. 2009, with the occurrence peaks still clearly centered before noon.

(i) For the large amplitudes above 100 pT, the limited data set of Li et al. 2009 unfortunately only very roughly characterizes the occurrence rates in the selected set of spatial bins, with huge random fluctuations, which evidently are related to the poor counting statistics. The occurrence rate in neighboring bins often differs by one order of magnitude or more. Within 10° from the equator (Figure 3a, rightmost panel), Li et al. 2009 still found peaks of occurrence rate between 7 and 8 MLT but more such random peaks occur between midnight and 6 MLT. This was not a new result of Li et al. 2009, as it corresponded to the known pattern that we describe in the manuscript as the traditional picture of postmidnight / dawnside chorus, see our refs 1 and 2, and lines 305-306. Our results for large amplitudes also show similar pattern of occurrence but with our higher resolution in latitude, we were able to localize it in our Figure 4e (and for even higher amplitudes in our Figure 4f) within 5° from the equator. Additionally, our much better counting statistics provide us with firm results without excessive random fluctuations and spurious peaks for this particular constraint.

Based on the comments of this reviewer we have now added a comparison of our results with the two papers referenced in the reviewer's report, exposing the strong disagreement of results of Li et al. 2009 with the results of Agapitov et al. 2013, and attempting to explain the differences compared to our present results (lines 406-419).

Response to Reviewer #3

We thank the reviewer for her/his time spent on the review, and for her/his constructive comments. The review report is quoted in *blue italics*, followed by our point-by-point responses. The line numbers refer to the revised manuscript with tracked changes.

The authors have performed an extensive statistical analysis of in-situ chorus and exohiss amplitude observations spanning approximately two solar cycles. The results show that the traditional picture of post-midnight and dawn chorus occurrence is incomplete. Only the very high amplitude (amplitudes larger than 100 pT) chorus waves exhibit higher occurrence in dawn and post-midnight. When accounting for lower amplitudes as well, the distribution of occurrence peaks around local noon.

Although the thresholds used for the wave detection have been carefully considered, some questions remain regarding the sample statistics.

Major comments

1) There is an issue of the sample size in each MLat bin. The RBSP observations are mostly equatorial (<=20 deg), while the Cluster spacecraft span the entire 0 to 60 deg range. Therefore, one can assume that the amount of data in each MLat bin introduces an imbalance. For the same reason, there are many more data points during the RBSP era (especially for low latitudes) than before. As the authors state under "methods", each Cluster dataset consists of ~2x10^7 data captures in the 2001-2020 time period, while each RBSP dataset consists of ~3x10^7 data captures during 2013-2019. This difference may introduce biases in the statistical analysis and the interpretation of the results.

We fully agree with the reviewer that our data set inhomogenously covers the analyzed intervals in time, latitude, and radial distance. The only nearly homogenously sampled variable is MLT. We also agree that the counting statistics is different in different latitudinal bins, which introduces different levels of random fluctuations but it should not introduce a systematic bias into the determined occurrence rates. As a part of our analysis procedure, we analyzed data from every spacecraft separately and we checked them for consistency when we processed data from the entire time interval of our analysis.

Following the reviewer's recommendation, we have added an extensive discussion of statistical significance and interpretation limits of our results on lines 420-469 and we have also verified that the results are consistent for different subintervals, as we describe it in the following response.

Have the authors checked the results using only Cluster during different time-periods, e.g., Solar cycle 23 vs 24 and/or during different solar cycle phases?

We have divided the analysis interval into four equally long consecutive parts, the first from 7 January 2001 till 5 November 2005, during the maximum and declining phase of Solar cycle 23, the second from 5 November 2005 till 4 September 2010, around the minimum of solar activity, the third from 4 September 2010 till 3 July 2015, during the raising phase and maximum of Solar cycle 24, and the fourth from 3 July 2015 till 30 April 2020, during the declining phase of Solar cycle 24 and the minimum of solar activity after it. The analysis is presented in Supplementary Figures 13 and 14, and in the text on lines 341-346, showing results consistent with our conclusions.

Complementary figures with the amount of data at each MLat vs MLT bin (similar to figure 3 in the manuscript) would be very helpful and would contribute substantially to the discussion of the results.

We have added these figures as the new Supplementary Figure 16. The discussion is added on lines 420-460.

Finally, the influence of the magnetospheric activity in terms of the Auroral Indices could possibly reveal if these features are driven (or not) by substorm activity. These figures could be included in the supplementary material.

We have added these figures as the new Supplementary Figure 15 and the text on lines 347-354, discussed further on lines 384-392.

2) Even though these results may significantly affect the outer radiation belt simulations, the authors do not discuss at all the physical mechanisms that lead to the occurrence peak at the local noon sector. The traditional picture of the peak occurrence in the post-midnight and dawn sectors is attributed to the injection of source electrons from the plasma sheet with a high temperature anisotropy. Is it possible that this temperature anisotropy is higher at local noon?.

We have added a discussion of possible sources of different types of chorus/exohiss on line 384-405.

Minor comments

1) Lines 22-23: It would be quite informative for the reader to mention here the frequency bands that chorus and hiss usually appear.

Added on lines 24-25.

2) Lines 12, 57 and 70-72: The sentence "90 years" is misleading. The Van Allen Probes mission begun in late 2012 and ended in mid-2019. This is almost 7 years of measurements. The existence of two spacecraft increases the amount of data (which are mentioned in the methods section) as well as the spatio-temporal coverage within the magnetosphere, but that does imply that one may simply multiply the duration of a mission with the number of satellites. Please mention the exact time-span of the observations instead of the "90 years" statement.

We have removed "of 90 years" from line 13 in the Abstract. We have also removed "total duration of 90 years of" from the Introduction, now line 59.

3) Line 63: Define lower band chorus.

Done on lines 65-66.

4) Line 79: …such as the one of the Van Allen Probes with the data from the high inclination orbit of Cluster,….

Wording modified on lines 84-85

5) Lines 83-84: The authors state: "We use the 3D measurement of the wave magnetic field to derive the total power spectral density". Does that mean that the authors estimate the power of the total magnetic field intensity?

Yes, this is correct. We have modified wording on lines 88-91.

*6) Figure 1: Generally, the solid lines are very hard to recognize. It would be better for the reader if the authors would plot the lines bold (thicker) and added a legend describing what they represent (e.g., 0.5*fce).*

We have replotted all the figures, including Figure 1, where we now use thicker lines, whose meaning is explained in the caption.

7) Line 131: A part of the plasmaspheric hiss shown in figure 1c corresponds to L values higher than 4.6 (left side of 1c). This is of course not surprising since the O'Brien model is empirical and thus expected to exhibit deviations from reality. Nevertheless, this raises a question of what portion of the following statistics is due to plasmaspheric hiss instead of chorus and exohiss and how much uncertainty the results have.

As a consequence of this reviewer's comment we have redone our analysis, removing all measurements at L below Lpp+1 from our analysis. This margin around the model plasmapause should take care of most of the random deviations of the model from reality. This is now described on lines 285-290 and discussed on lines 420-424.

8) Lines 142-143: Is this a linear or logarithmic averaging? Since the values span several orders of magnitude one could assume that a linear averaging favors the higher values.

The Van Allen Probes onboard procedure used an arithmetical average to combine the initial frequency bins from the FFT into the resulting 65 channels. The complex amplitudes in these initial FFT bins were only internal values of this onboard procedure and did not make it to the telemetry. Only the averaged spectral matrices were downlinked and archived in the resulting data set. The variations by several orders of magnitude within this data set results from accumulation of a large number of measurements over time. We have reworded the text on lines 521-529.

9) Lines 145-146: Is kappa the same for RBSP and Cluster? If this is linked to the resolution of the data, I would assume it is not the same; nevertheless, only one correction factor is mentioned here. Please clarify.

As a consequence of this comments, we have redone the analysis and we now more accurately capture the difference between Cluster and RBSP onboard analysis. We describe our procedure in detail in two new paragraphs of the Methods section on lines 576-645, with new Supplementary Figures 1-3 and also with adding Cluster results to Figure 3.

10) Line 161: There is only figure 1 and not figure 1a in the supplementary material.

This is now corrected on line 224 for Supplementary Figures 4 and 6 (after the insertion of new Supplementary Figures 1-3)

11) Lines 336-337: If the 11 RE is a hard threshold, how the authors can be sure that the data used are always inside the magnetopause?

In the previous version of our analysis procedure, we assumed that the hard outer limit of 11 R_E would be sufficient to exclude most of the measurements in the magnetosheath. As a consequence of this reviewer's comment, we recognized that this might have introduced a bias to our results. We have

redesigned our analysis procedure and rerun the analysis. We now include an additional limit in our procedure to include only the measurements inside the model magnetopause of Shue et al. 1998. We also modified the analysis to exclude the measurements where the frequency range of the instrument didn't fully extend over the analyzed frequency interval. This is described on lines 538-548 and 566-575 in the Methods section. With the updated procedure, the size of the dataset slightly decreased and the average number of instrumental frequency channels in the analyzed frequency range increased. Consequently, numerical values for probabilities in Section 1 also slightly changed, as did Figure 4, but the conclusions of our study remain the same.

12) Lines 348-371: This is confusing. Are the measurements indeed cross-calibrated to obtain the results shown in figure 8 or do the authors show the comparison of the native measurements suggesting that the divergence of the two mission measurements are really close anyway? If the latter is true, then the term "inter-calibration" should not be used here.

By intercalibration we meant here the calibration of noise thresholds to the same probability of the false positive detection. These thresholds vary as a function of frequency, with time and naturally between different spacecraft. We have therefore replaced this paragraph by a thorough description of our detection threshold procedure on lines 576-645 and with new Supplementary Figures 1-3.

Response to Reviewer #4

We thank the reviewer for her/his time spent on co-review our manuscript, and for her/his contribution to the constructive comments of one of the reviewers whom we responded above.

Response to Reviewer #1

We thank again the reviewer for her/his time spent on the review. The review report is quoted in *blue italics*, followed by our response.

Second Review of "Prevailing Dayside Occurrence of Lower Band Whistler Mode Chorus and Exohiss" by Santolik, Kolmasova, Taubenschuss and Hanzelka

I find the paper acceptable for publication if the authors give credit to others who have reported similar chorus features. I have no further comments.

Based on a new set of comments of reviewer #2 we have prepared the second major revision of the manuscript. We have conserved all changes, which resulted from all previous reviewers' comments, and which we introduced into the previous revision of the manuscript, while we have removed the details of the procedure to determine the noise thresholds. This procedure, the resulting data set of noise thresholds, and the entire underlying data set of lower band chorus/exohiss amplitudes, which serves as the source data set for the present manuscript, are now published in Scientific Data (https://doi.org/10.1038/s41597-025-05531-6). In this second revised version we made sure to give credit to others who have reported similar chorus features.

Response to Reviewer #2

We thank the reviewer for her/his time spent on the review. The review report is quoted in *blue italics,* followed by our point-by-point responses. The line numbers refer to the revised manuscript with tracked changes.

As a result of the additional comments of this reviewer, we have changed the focus of the paper, while conserving the results of our analysis. In this second major revision of the manuscript, we made sure to conserve all changes, which resulted from all previous reviewers' comments, and which we introduced into the previous revision of the manuscript, while we have removed the details of the procedure to determine the noise thresholds. This procedure, the resulting data set of noise thresholds, and the entire underlying data set of lower band chorus/exohiss amplitudes, which serves as the source data set for the present manuscript, are now published in Scientific Data (https://doi.org/10.1038/s41597-025-05531-6).

After carefully reading the revised manuscript and the Response to my previous comments, I regret to write that my position concerning this manuscript is unchanged. The main results of the present manuscript (emphasized in the Title and Abstract) concern the global features of chorus wave occurrences as a function of MLT (local time), wave amplitude, and latitude, and they were already discovered 15 years ago by Li et al. (2009). The "prevailing dayside occurrence" of moderate amplitude chorus waves (emphasized in both the Title and Abstract of the present manuscript) was first discovered by Li et al. (2009) and confirmed by Agapitov et al. (2013). The shift of chorus wave occurrences toward the postmidnight sector for wave amplitudes above 100 pT (also emphasized in the Abstract of the present manuscript) was also first discovered by Li et al. (2009). These important discoveries justified the publication of the results of Li et al. (2009) in Geophysical Research Letters (a journal roughly equivalent to PRL in the limited field of geophysics).

We changed the title and we rewrote the abstract, emphasizing new results of our study.

In the revised manuscript, the differences between the details within these global features of chorus wave occurrences, like in this or that individual MLT bin, and the different numbers of years of data used, and the details of the different data analysis procedures used in each study, are extensively discussed. But at the same time, the strong similarities of the main, global features of chorus wave occurrences shown in the present Figure 4 with the previous results in Figure 3 of Li et al. (2009) and Figure 3(b,f) of Agapitov et al. (2013) are not acknowledged. This can easily give the misleading impression that the main results of the present manuscript (on the global features of chorus wave occurrences) are new, which is not exact.

We now acknowledge (lines 278-280, 300-308) that the main global features of chorus wave occurrences shown in the present Figure 2 (renumbered from Figure 4 in the previous version) are similar to the previous results in Figure 3 of Li et al. (2009) and Figure 3(b,f) of Agapitov et al. (2013).

The present study, performed 15 years later, provides a more precise knowledge of the very fine details of the chorus wave occurrence distribution in each individual MLT (local time) bin than in Li et al. (2009) or Agapitov et al. (2013), but this incremental improvement is clearly insufficient to justify a publication in Nature Communications. It does not provide a significant improvement to our knowledge of the physical processes leading to chorus wave generation.

We believe that the newly emphasized probability distribution function of chorus amplitudes (Figure 1) as the basis of our results in Figure 2 and the corresponding temporal distribution of delays between detection with the same set of amplitude thresholds (Figure 3) provides a significant improvement to our knowledge of the physical processes leading to chorus wave generation, which can stimulate new development in this field.

Finally, for simulations of the effects of chorus waves on the dynamics of radiation belt electrons (the "critical role" of chorus waves mentioned in lines 11-12 of the Abstract), the only important parameter is the CHORUS WAVE POWER DISTRIBUTION (e.g., see Thorne et al. 2013, Wong et al. 2024 doi:10.1029/2023JA031607) and NOT the CHORUS WAVE OCCURRENCE DISTRIBUTION discussed in the present manuscript. Various models of this chorus wave power distribution have been provided by Meredith et al. (2012, 2020) and Agapitov et al. (2018) based on the same data from the Van Allen Probes and Cluster missions. These three recent studies did not examine anymore the "wave occurrence distribution" in isolation, contrary to what is done in the present manuscript, simply because this "wave occurrence distribution", alone, is known to be unimportant for simulations of the dynamics of radiation belt electrons.

All the above mentioned papers are now on our list of references, with proper references in the text. We fully agree with the reviewer and with the conclusions or prerequisites of these papers, that the most important parameter for the quasilinear modeling (besides the wave normal distribution) is the chorus wave power distribution. We present it now in Figure 1. Figure 2 (former Figure 4) is only another representation of this distribution, showing occurrence percentages with different thresholds for wave power. This Figure is coupled to our new results on temporal properties of the corresponding waves shown in Figure 3, showing the spatial counterparts of these results.

Therefore, the present manuscript deserves to be published in a more specialized journal, like Journal of Geophysical Research (Space Physics).

(1) The three main results of the present manuscript (presented in Figure 4), concerning the global features of chorus wave occurrences as a function of MLT (local time), wave amplitude, and latitude, presented in Figure 4, were already discovered 15 years go by Li et al. (2009).

- First, it is clear in Figures 3(a) of Li et al (2009) for moderate amplitude waves (Bw=10-30 pT) at Latitudes < 10 deg that the peak occurrence rate patch (yellow-red) is located at 7-13 MLT at L > 7 and at 8-17 MLT at L = 5-7, i.e., on the dayside.

It is also clear in Figures 3(b) of Li et al (2009) for moderate amplitude waves (Bw=10-30 pT) at Latitudes 10-25 deg that the peak occurrence rate patch (yellow-red) is also located on the dayside, at 8-13 MLT at L > 7 and at 7-17 MLT at L = 5-7.

- Second, it is also quite clear from Figure 3(a) of Li et al. (2009) that when wave amplitude Bw increases from Bw=10-30 pT to Bw = 30-100 pT to Bw > 100 pT, the peak occurrence rate patch (yellowred) at L = 5-7 moves from the dayside (8-17 MLT) to the postmidnight (midnight-to-dawn) sector (0-8 MLT) at la[illegible]tudes < 10 deg.

- Third, comparing Figures 3(a) and 3(b) from Li et al (2009), it is clear that the shift of occurrence rates from dayside to the postmidnight (midnight-to-dawn) sector present at latitudes < 10 degrees, is not present at higher latitudes > 10 degrees.

These three major discoveries from Li et al. (2009) on the global features of chorus wave occurrences as a function of MLT, wave amplitude, and latitude, are the SAME as the THREE MAIN RESULTS of the present manuscript (shown in Figure 4 and noted in the Title itself and the Abstract).

We now acknowledge (lines 278-280, 300-308) that the main features of chorus wave occurrences shown in the present Figure 2 (renumbered from Figure 4 in the previous version) are globally similar to the previous results in Figure 3 of Li et al. (2009). We have changed the focus of the paper, while conserving the results of our analysis, so that the main emphasis is now on the results, which are different from previous work. We have changed the title and we rewrote the abstract, emphasizing new results of our study.

Figures 3(b,f) from Agapitov et al. (2013) similarly show a broad peak of chorus wave occurrences on the dayside (at 9-19 MLT) at L = 5-8 when considering all such waves measured by Cluster, i.e., mainly waves of moderate amplitudes. As shown in Figures 3(a,e,i) of Agapitov et al. (2013), the corresponding average amplitudes of such dayside chorus waves in their Figures 3(b,f) are Bw = 10-20 pT, far above the instrument noise level (contrary to the statements in lines 385-386 of the revised manuscript) and similar to the "moderate amplitude waves" of the present manuscript and of Li et al. (2009).

We now acknowledge (lines 278-280, 300-308) that the main global features of chorus wave occurrences shown in the present Figure 2 (renumbered from Figure 4 in the previous version) are similar to the previous results in Figure 3 of Li et al. (2009) and Figure 3(b,f) of Agapitov et al. (2013).

We have removed all statements about the presence of instrumental noise in the results of Agapitov et al. (2013).

(2) The manuscript (especially lines 379-392), as well as the response, are confusing. They focus on some fine details in the figures of Li et al. (2009), on some presumed limitations of the datasets of Li et al. (2009) or Agapitov et al. (2013), and on the obtained precise percentage level of chorus wave occurrences (this level, however, is known to vary in time and over different solar cycles, so that one cannot expect to get the same level with different datasets covering different time spans). There are also instances where previous works are strongly minimized without supporting evidence (lines 379 and 385-386).

We have removed all statements about the limitations of the datasets of Li et al. (2009) or Agapitov et al. (2013), although we still believe that the THEMIS filter bank data set is limited, especially in the number of analyzed components and in the number of frequency channels. We also removed the discussion of differences of our results with respect to those of Li et al. (2009) or Agapitov et al. (2013). We have also removed the instances where previous works might have seem to be minimized without supporting evidence, although this was not our intention.

We also discuss the variation of our results over different solar cycles covered by our data set (lines 212-217).

However, the main facts remain: THE MAIN, GLOBAL FEATURES ARE THE SAME AS IN LI ET AL. (2009) AND AGAPITOV ET AL. (2013). At low latitudes, both the "prevailing dayside occurrence" (8-17 MLT) of moderate-amplitude chorus waves and the shift of this broad peak of chorus wave occurrences to the postmidnight (midnight-to-dawn) sector (0-8 MLT) at high wave amplitude were already discovered by Li et al. (2009). At high latitudes, the absence of this MLT shift of the peak occurrences from 8-17 MLT to 0-8 MLT as the wave amplitude increases, was also discovered by Li et al (2009).

The dayside (9-19 MLT) peak of occurrence of moderate-amplitude chorus waves at L = 5-8 was also confirmed by Agapitov et al. (2013) at both low and high latitudes.

We now acknowledge (lines 278-280, 300-308) that the main global features of chorus wave occurrences shown in the present Figure 2 (renumbered from Figure 4 in the previous version) are similar to the previous results of Li et al. (2009) and Agapitov et al. (2013).

Of course, the more limited statistics of Li et al. (2009) or Agapîtov et al. (2013) did not allow them to provide a very precise MLT distribution of occurrences in each individual MLT bin, as done in the present manuscript. But these improvements provided in the present manuscript are merely incremental improvements, compared with the important discoveries of Li et al. (2009), and such incremental improvements are clearly insufficient to justify a publication in a high-level journal like Nature Communications.

We believe that the newly emphasized probability distribution function of chorus amplitudes (Figure 1) as the basis of our results in Figure 2 and the corresponding temporal distribution of delays between detection with the same set of amplitude thresholds (Figure 3) provides a significant improvement to our knowledge of the physical processes leading to chorus wave generation, which can stimulate new development in this field.

(3) Lines 54-56, it is written that "Another simplification is that intervals with instrumental noise are simply included in the long term averages, as none of the existing models properly introduces detection thresholds nor the occurrence rates of chorus and exohiss". This unjustified statement should be

suppressed or seriously reformulated, because it is false. Most existing chorus wave models (e.g., from Li et al., (2009), Meredith et al. (2012, 2020), Agapitov et al. (2013, 2018)) properly incorporated occurrence rates and used a detection threshold in wave amplitude at 1-10 pT and/or additional thresholds leading to a high signal-to-noise ratio.

We removed this statement.

For instance, Meredith et al. (2012) noted the following about their chorus wave model (in their Section 6): "Differences in sensitivity between the instruments can affect the average intensities but are limited to very low intensity levels when the spectral intensity is below the threshold for detection in one or more of the instruments. The effect is negligible when the wave power is well above the threshold for detection for all of the instruments, which roughly equates to wave power above 1 pT^2. Since chorus wave power below 1 pT^2 is insignificant and has an insignificant affect on diffusion rates our model captures all of the important chorus wave power."

The paper of Meredith et al. (2012) is on our list of references and properly introduced in the text. We agree with this statement, which is consistent with the text of the last revision of our paper.

(4) Lines 262-263: "A very important aspect of our study is that we increased the statistical significance of our results by combining the data of the Cluster and Van Allen Probes missions." This "very important aspect of the present study" is not new at all: Agapitov et al. (2018) already combined the data of the Van Allen Probes and Cluster missions to provide a model of time-averaged chorus wave power, naturally incorporating chorus wave occurrences.

We have removed this statement.

(5) Using an empirical plasmapause model (as in the present study) is not very accurate for determining the plasmapause and the L-shell domain where chorus waves are presumed to be present at each time. It would be much better to use actual plasmapause crossings available from Van Allen Probes density measurements.

We agree with the reviewer that it would be better to use the actual plasmapause crossings (although new results of D. Hartley suggest that excluding the orbits where the plasmapause density gradient is not properly identified can bias the overall results). However, as our study concerns also latitudes above the Van Allen Probes range, the empirical plasmapause model is the only possibility to conserve the consistency between Van Allen Probes and Cluster data sets. We discuss the usage of the model on lines 133-142 and 415-421.

(6) Lines 264-269. The authors only consider chorus waves inside a domain comprised between the model plasmapause plus 1 Earth's radius (RE), and the field line at an equatorial distance of 6 RE. This suggests that they exclude many quiet times (i.e., moderate amplitude) chorus waves located at 5.5-7.0 RE, potentially leading to an important bias in the provided MLT distribution of moderate amplitude chorus waves in the outer radiation belt.

We actually consider chorus waves inside a domain comprised between the model plasmapause plus 1 Earth's radius (R_E), and the model plasmapause plus 6 R_E, so the quiet time chorus is not excluded.

Response to Reviewer #3

We thank the reviewer for her/his time spent on the review. The review report is quoted in *blue italics*. followed by our response.

Dear authors,

thank you for the thoroughly revised manuscript and your detailed responses to the reviewers' comments.

I now consider the manuscript acceptable for publication.

Ioannis A. Daglis

Based on a new set of comments of reviewer #2 we have prepared the second major revision of the manuscript. We have conserved all changes, which resulted from all previous reviewers' comments, and which we introduced into the previous revision of the manuscript, while we have removed the details of the procedure to determine the noise thresholds. This procedure, the resulting data set of noise thresholds, and the entire underlying data set of lower band chorus/exohiss amplitudes, which serves as the source data set for the present manuscript, are now published in Scientific Data (https://doi.org/10.1038/s41597-025-05531-6).

Response to Reviewer #4

I co-reviewed this manuscript with one of the reviewers who provided the listed reports. This is part of the Nature Communications initiative to facilitate training in peer review and to provide appropriate recognition for Early Career Researchers who co-review manuscripts.

We thank the reviewer for her/his time spent on co-reviewing our manuscript, and for her/his contribution to the constructive comments of one of the reviewers whom we responded above.

RESPONSE TO REVIEWER COMMENTS

on Nature Communications manuscript NCOMMS-24-13847B-Z

„Spatiotemporal Patterns of Lower-Band Whistler Mode Waves in the Earth's Magnetosphere"

by O. Santolík, I. Kolmašová, U. Taubenschuss, M. Hanzelka

Reviewer #2 (Remarks to the Author):

Overall, this very significantly revised version of the original paper provides much more useful and interesting results on the chorus wave power distribution and on time delays between chorus wave power occurrences at high wave power. These distribution should be useful for better modeling the effects on electron fluxes and understanding the generation of chorus waves. However, several important points should be improved before a possible publication.

We thank the reviewer for her/his insightful comments and suggestions, which helped us to improve the manuscript. We took all of them into account while preparing the revised version. The line numbers in our point-by-point responses refer to the document with tracked modifications.

The most important problem is with new Figure 3, where the plotted time delays may not always be representative of actual delays (suggestions for improvements are provided below).

We have redone Figure 3 following the reviewer's comments (responses to specific comments are given below)

The suggested impact on long-term simulations of radiation belts should also be discussed more accurately.

We have modified this discussion according to the reviewer's suggestions (responses to specific comments are given below).

1. New Figure 1, showing PDFs of chorus amplitude, is an important addition for undestanding chorus wave generation and propagation mechanisms. You could further note in the text (near Line 108) that the the long-term average (white line) provided in Panel (e) should be used in long-term radiation belt quasi-linear simulation and that, although this provided long-term average roughly agrees with previous statistics (Agapitov et al. 2018), it is more accurate and available up to higher latitudes thanks to the larger dataset used. This distribution in Panel (e) may also account for the apparent presence of significant chorus wave ducting in the noon sector recently inferred from ELFIN measurements of the precipitating-to-trapped electron flux ratio (Zhou et al. 2025 https://doi.org/10.1029/2025JA034117).

The relevance of the long-term average (white line) for long-term radiation belt quasi-linear simulation is now noted on line 108-109.

Comparison with (Agapitov et al. 2018) and (Zhou et al. 2025) is now given in the Discussion section on lines 399-403.

2. I have some concerns about new Figure 3 and suggestions of improvements.

As concerns Figure 3, the Authors should note in the Main text that based on the typical orbital speed, spacecraft should traverse the chorus source region in 100-200 s for typical "chorus source region" transverse sizes (at 0.5 correlation) of 400-800 km determined using Van Allen Probes data (Agapitov et al. 2021 https://doi.org/10.1029/2020JA028998, Z. Zhang et al. 2021 https://doi.org/10.1029/2021JA029569).

Results of Agapitov et al. 2021 and Zhang et al. 2021 are now discussed on lines 414-427 in the Discussion section.

This implies that the present observations are usually not independent for time delays less than 200 s, which probably explain the power-law shape in Figure 3 for delays less than 200 s. Agapitov et al. 2021 (https://doi.org/10.1029/2020JA028998) further noted that the typical wider "chorus active region" size (defined as the maximal distance at which chorus waves are observed simultaneously in at least 50% of the observations with squared amplitudes higher than 9 pT^2, see their Fig.7d) is 4000-8000 km at 8-14 MLT in the transverse direction, corresponding to time delays of 15-35 min. This implies the presence of additional correlations between spacecraft observations of chorus waves, up to delays of 2000 s. This order of value is in good agreement with the range 10-2000 s of time delays in Figure 3 where the power-law slope remains constant.

The transversal component of Van Allen Probes and Cluster orbital velocities can be found in a large range of values for different orbits and locations, but typically, the interval 2-3 km/s covers the main part of the possible range. The traversal time through the wider "chorus active region" of 4000-8000 km can therefore explain the dayside observations of a characteristic change in the probability density function of delays, occurring around 30 min. We also keep open the possibility that this change reflects temporal rather than spatial properties of the larger global chorus source region. Lower scales of chorus sources (below 1000 km), which can be found in literature do not seem to play a visible role in the delay statistics but we still mention them in the discussion for completeness. We added new paragraphs in the Discussion section on lines 404-427.

Above 2000 s delays, the negative slope often decreases before increasing again above 2 10^4 s in Figure 3. The Authors already wrote about the spatial chorus correlation

(Lines 522-524 in Methods Section), but this important point should also be mentioned in the main text to explain Figure 3 based on typical chorus source region and chorus active region sizes determined using the same Van Allen Probes dataset as in the present work.

This is now mentioned in the Discussion section on lines 404-427.

However, for time delays longer than about 10^4 s, a Van Allen Probe has already travelled far away from its initial (L,MLT) region of measurements and the recurrence periods between consecutive measurements at a same L or at a same (L,MLT) should start to play an important role in the distribution of time delays. Such recurrences should introduce additional correlations between measurements and might account for the more rough power-law shape above 10^4 s. Cluster spacecraft have longer orbital periods but more inclined orbits, so that recurrences at a same L or (L,latitude) should also start to play a role above 10^4 s. The presence of such recurrences at long time delays suggests that the results in Figure 3 should be taken with caution above 10^4 s. They likely do not fairly represent the distribution of actual time delays at a given, fixed L or (L,MLT) or (L, latitude).

Therefore, I would suggest to stop the curves in Figure 3 as dashed lines at 10^4 s.

We now stop all the panels of Figure 3 and the newly added Figure 4 at 16,200 s, which corresponds to one half of the orbital period of Van Allen Probes.

It would also be important and useful to plot additional panels in Figure 3, showing the same distribution of delays in fixed (L, MLT) ranges (say +/- 0.5 R_E in L and +/- 1 MLT) at 11-13 MLT and 0-2 MLT, above 10^3 to 10^5 pT^2 (2 x 3 = 6 additional panels).

This could provide more useful distribution of delays for radiation belt simulations.

A new Figure 4 is added in the revised version according to this reviewer's suggestion. The corresponding newly added text is on lines 294-321.

You may consider keeping only these new panels instead of the present panels, because the present panels are less easy to interpret and probably provide less reliable distributions of delays in a given L or (L,MLT) region (the latter are the most interesting for simulations and for understanding chorus generation mechanisms).

We prefer to keep both Figure 3 and the new Figure 4. They provide complementary views of the delay statistics, both global and localized to important regions. Besides having much weaker statistics, the localized approach is not free of interpretation problems, either. The Figure below shows a wide range of traversal durations for the two selected regions, which is necessarily reflected by maximum possible delays for separate orbits. This problem is briefly described on lines 307-314.

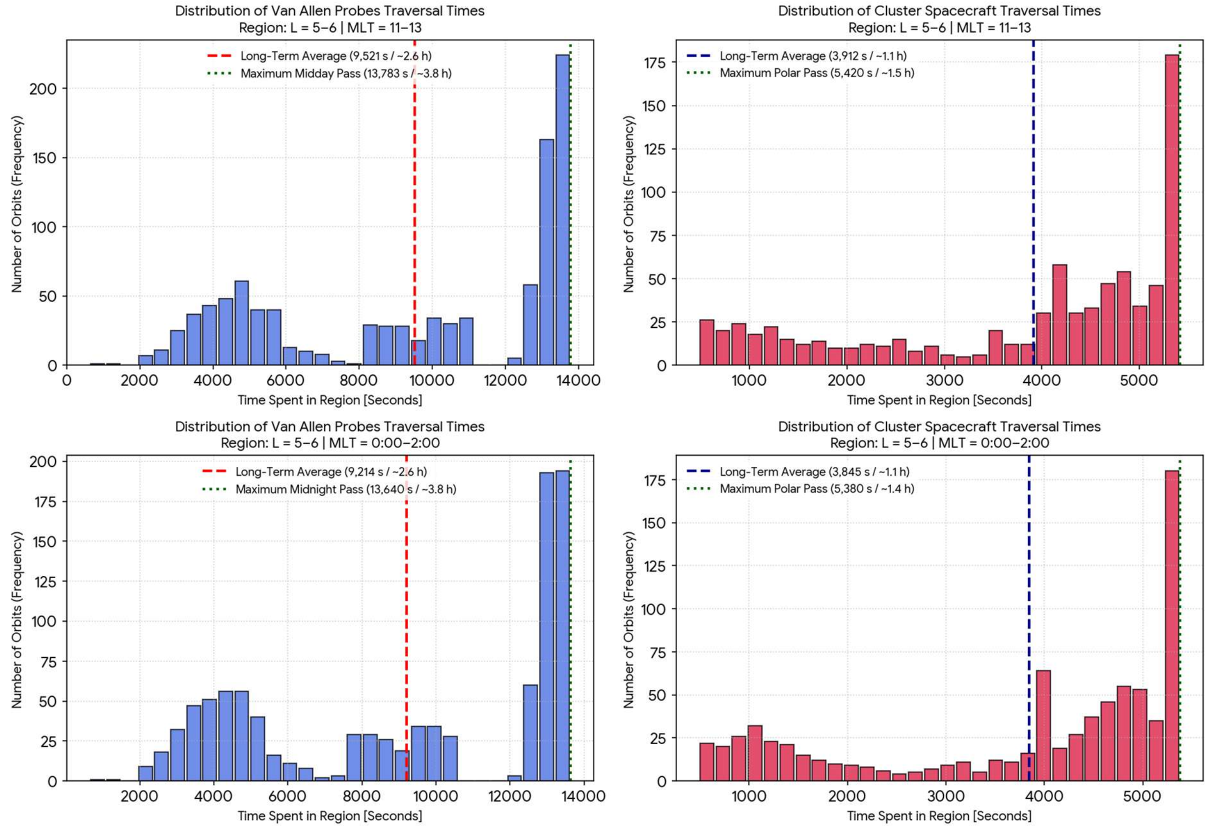

3. The statements in Lines 380-382 and 390-395 and are excessive and should be seriously amended. The present dataset consists of 0.5-s-averaged wave power (VAP) or 4-s-averaged power (Cluster). Actual chorus wave elements mainly consist of trains of relatively short intense wave packets lasting less than 10-20 wave periods (0.005-0.1 s), with a roughly power-law shape PDF of wave packet power at 0.1-1 nT (X. Zhang et al. 2021 https://doi.org/10.1029/2021JA029330).

These statements are now substantially amended and put into the context of the chorus subpackets on lines 487-502. The work of X. Zhang et al. 2021 is now referenced in the context of previous work on this subject on lines 499 and 500.

Therefore, intense wave packets of 10^4 pT^2 or 10^5 pT^2 should be much more frequent than 0.5-s or 4-s bursts of the same power at a given L, and occur after much shorter time delays than in Figure 3. In other words, wave packet variability, including wave phase and amplitude variability and radial variability, may still allow quasi-linear theory to remain applicable (Artemyev et al 2025 https://doi.org/10.1007/s11214-025-01144-y, Hanzelka et al. 2025 https://doi.org/10.1029/2025GL116883).

It was not our intention to question the validity of the quasilinear theory, we therefore fully agree with the reviewer. This is now made clear on lines 499-502. We also agree that the large instantaneous amplitudes should occur more often than the large average amplitudes (lines 499-501).

Reviewer #3 (Remarks to the Author):

Although the study is primarily a statistical characterization of wave amplitude distributions and spatiotemporal patterns and does not directly study chorus generation mechanisms at the microphysical level, it nevertheless advance knowledge relevant to chorus generation physics through several of its results.

We thank the reviewer for her/his insightful comments and suggestions, which helped us to improve the manuscript. We took all of them into account while preparing the revised version. The line numbers in our point-by-point responses refer to the document with tracked modifications.

1. A major new finding is the clear separation of two regimes as a function of wave amplitude threshold:

- Low-amplitude waves occur on the dayside, noon-centred, across all latitudes up to ±50°.

- While for high-amplitude waves the occurrence shifts sharply to the post-midnight equatorial region (low MLat).

This amplitude-dependent shift, documented quantitatively for the first time with this statistical weight (~26 years of data), directly constrains the two distinct generation populations described by the authors.

Prior work (THEMIS, Li et al. 2009; Cluster, Agapitov et al. 2013) had lower coverage and noted a dayside peak, but could not resolve this amplitude-dependent transition from dayside to post-midnight dominance with the same precision.

We now note this with the suggested references in the revised version on lines 324-330.

2. The discovery that time delays between detections follow a power-law (not Poisson) distribution is a genuinely new physical constraint. A Poisson distribution would indicate independent, memoryless events — consistent with steady or quasi-random linear growth. A power law reflects clustering across multiple timescales, implying that wave generation events are not independent but mutually correlated — consistent with bursty, injection-driven cyclotron instability where one substorm injection triggers a

wave burst, whose envelope modulates subsequent bursts. Previous studies noted the bursty character qualitatively; this is the first large-scale statistical quantification of the inter-event time distribution and its dependence on amplitude.

We have added an excerpt from this description on lines 336-339.

3. The substorm–chorus link has been confirmed and extended.

4. The rising occurrence of low-amplitude waves on the dayside near solar maxima, points to a solar wind–controlled generation pathway for the dayside wave population that is distinct from the substorm-driven post-midnight pathway.

Comments on the substorm origin of postmidnight chorus are given on lines 353-361, and solar wind related origin of the dayside waves is mentioned on lines 384-394.

However, there are also some methodological and conceptual shortcomings. In particular:

1. The most significant scientific limitation - acknowledged by the authors - is that chorus and exohiss are analysed together as a single population throughout the study. It is a fundamental problem, because the two wave types have very different generation mechanisms. Binning them together blurs physically distinct phenomena.

We, indeed, acknowledge this limitation but we see no way to remove it with the existing data set. As we note on lines 447-450, high-resolution spacecraft measurements of whistler-mode waves are usually triggered by high amplitude signals, and therefore the currently available collections of spacecraft measurements do not allow us to build large unbiased databases of wave amplitudes separately for chorus and exohiss. The onboard analyzed spectral data that we use in this work are regularly sampled and therefore unbiased in this sense, but they do not allow us to distinguish chorus from exohiss because of their time/frequency averaging. We attempt to mitigate this problem by showing examples of high-resolution spectrograms captured in the regions of occurrence of the two populations of the lower band waves in Figure 5 and in Supplementary Figure 8. These examples demonstrate that both chorus and less structured hissy emissions can be observed in both distinct populations of lower band waves (post-midnight and dayside), and that there is probably a smooth transition between these spectral forms.

2. The dataset is heavily non-uniform in magnetic latitude coverage. The authors note this but claim the statistics remain adequate. However, for heavily constrained subsets (e.g., intense waves during specific solar cycle phases at high latitudes), the coverage

degrades severely, causing large random fluctuations that the authors acknowledge as a real limitation.

Indeed, we also acknowledge this limitation, which is clearly demonstrated in Supplementary Figure 7a and discussed in the Methods section on lines 597-620. The combination of two spacecraft missions at different orbits (equatorial orbit of the Van Allen Probes and high inclination orbit of Cluster) is at the origin of this disbalance. We believe that, with this largest available consistent data set covering a large range of latitudes, we have no other choice than accepting this limitation. Thanks to the Cluster orbit, the coverage is still large up to 50 degrees of magnetic latitude. It is true that with further constraints on the data, for example according to specific time intervals during the Solar cycle (as in Supplementary Figure 7d), the statistics become poor at high latitudes, as we acknowledge on lines 615-620, but there is no solution to this problem with the currently available data sets.

3. The L parameter and the plasmapause boundary are calculated using a pure dipole magnetic field, which is a recognised oversimplification.

We again agree with the reviewer. When designing the parameters of the cleaned and processed data set (published as doi:10.1038/s41597-025-05531-6) that we use in the present study, we chose the simple dipole model over the more accurate parametrization by L* mainly because of the simplicity of its interpretation. With the average 0.74-R_E uncertainty (recognized on line 530) of the plasmapause model of O'Brien & Moldwin (2003), we believe that dipole L provides us with an appropriate parametrization for the purpose of this study.

4. The Van Allen Probes operated during the declining phase of Solar Cycle 24 only (~2012–2019), whereas the Cluster mission spans Solar Cycles 23 and 24. The two datasets therefore sample systematically different solar activity distributions.

We also agree with this statement. The timing of the two missions is defining the distribution of solar wind and geomagnetic parameters they encountered but there is no reasonable way to change it unless we limit the Cluster data set to the overlapping time interval for the two missions. This was done to validate the combined data set (doi:10.1038/s41597-025-05531-6, figure 13) under comparable external conditions. Extending then the Cluster data to a larger time interval enables us to sample a wider range of geomagnetic activity indices.

Nevertheless, the manuscript merits publication despite its shortcomings.
